# Contamination of tea leaves by anthraquinone: The atmosphere as a possible source

**DOI:** 10.1007/s13280-023-01858-9

**Published:** 2023-04-28

**Authors:** Cathy W. Y. Li, Stacy Walters, Jean-François Müller, John Orlando, Guy P. Brasseur

**Affiliations:** 1 Environmental Modelling Group, Max Planck Institute for Meteorology, Bundesstrasse 53, 20146 Hamburg, Germany; 2 Atmospheric Chemistry Observations & Modeling Lab, National Center for Atmospheric Research, P.O. Box 3000, Boulder, CO 80307 USA; 3 Department “Sources and Sinks of Atmospheric Constituents”, Royal Belgian Institute for Space Aeronomy, Ringlaan 3 Avenue Circulaire, 1180 Brussels, Belgium

**Keywords:** Air pollution, Anthraquinone, Atmospheric transport, Food contamination, Pesticide residue limits, Tea

## Abstract

The detection of anthraquinone in tea leaves has raised concerns due to a potential health risk associated with this species. This led the European Union to impose a maximum residue limit (MRL) of 0.02 mg/kg for anthraquinone in dried tea leaves. As atmospheric contamination has been identified as one of the possible sources of anthraquinone residue, this study investigates the contamination resulting from the deposition of atmospheric anthraquinone using a global chemical transport model that accounts for the emission, atmospheric transport, chemical transformation, and deposition of anthraquinone on the surface. The largest contribution to the global atmospheric budget of anthraquinone is from residential combustion followed by the secondary formation from oxidation of anthracene. Simulations suggest that atmospheric anthraquinone deposition could be a substantial source of the anthraquinone found on tea leaves in several tea-producing regions, especially near highly industrialized and populated areas of southern and eastern Asia. The high level of anthraquinone deposition in these areas may result in residues in tea products exceeding the EU MRL. Additional contamination could also result from local tea production operations.

**Supplementary Information:**

The online version contains supplementary material available at 10.1007/s13280-023-01858-9.

## Introduction

Anthraquinone (C_14_H_8_O_2_, noted AQ), an oxygenated polycyclic aromatic hydrocarbon (OPAH), is present in the atmosphere with concentrations varying greatly with geographic location, from less than 1 ng/m^3^ in remote regions to more than 100 ng/m^3^ near pollution sources. It is released primarily by combustion processes (e.g., coal and biofuel burning, automobile exhaust, biomass burning) and from waste streams (International Agency for Research on Cancer 2012; Lin et al. 2015; Lim et al. 2021; Pereira et al. 2021), and it is used as a bird repellant and in the production of cardboard. It is also formed in situ by the oxidation of anthracene (C_14_H_10_, noted ANT), a polycyclic aromatic hydrocarbon (PAH) also detected in vehicle exhaust, produced by other combustion sources, and observed in the vicinity of waste sites. It has been suggested that AQ is potentially mutagenic and carcinogenic. Therefore, this substance could pose environmental health risks (Shukla et al. 2017), although some recent studies did not identify adverse health effects for populations consuming tea (Yang et al. 2022). In fact, according to toxicological studies, the toxicity of PAH-derived quinones is believed to exceed that of their parent PAHs, due to their higher efficiency to form DNA adducts and higher bio-accessibility (Bolton et al. 2000; Yu 2002; Lammel et al. 2020). Quinones generate reactive oxygenated species (ROS) through their redox cycles with the corresponding semiquinones, which can damage cell structures (e. g., oxidative damage to DNA and proteins) and lead, for example, to adverse effects on vascular and pulmonary cells (Ohkuma et al. 2001; Kubátová et al. 2006; Wei et al. 2010; Misaki et al. 2016). ROS are suspected to be a cause of carcinogenesis and favor apoptosis, the programmed death of cells in multicellular organisms (Redza-Dutordoir and Averill-Bates 2016).

Anthraquinone residues found in food substances, for example in tea leaves, therefore pose potential health risks. In fact, laboratory measurements have shown that tea leaves imported from Asia and distributed in Europe often contain excess concentrations of anthraquinone (European Food Safety Authority 2018). According to a 2016 European Union report on pesticide residues in food from the European Food Safety Authority, anthraquinone residues are found to exceed the Maximum Residue Limit (MRL) in 115 of 1016 tea samples (European Food Safety Authority 2018). The current MRL for anthraquinone in tea products is 0.02 mg of the substance per kg of dry tea leaves^1^ (The European Parliament and the Council of the European Union 2005). This compound has been detected in tea samples from India and China (European Food Safety Authority 2018). The largest reported exceedance corresponds to residues of 0.37 mg/kg (European Food Safety Authority 2018).

The production of black tea in tropical and subtropical areas involves hand-plucking of leaves from the top of the tea plant every 4 to 15 days as well as several successive operations: withering (with reduction of leaf moisture from typically 75 to 45%), rolling (breaking down of internal cell structure to release enzymes), oxidation (fermentation at a controlled temperature to produce color and flavor), drying (further reduction in moisture content to 2–3% at high temperature), packaging and commercial dissemination. Some of these operations, such as those during which coal and wood are burnt as fuel, could be a local source of anthraquinone in processed tea products.

Several studies investigating the source of anthraquinone contamination in tea products concluded that endogenous formation of anthraquinone in the tea plant is unlikely (Romanotto and Gassert 2017). Rather, they suggest that outdated tea production and packaging processes (such as using traditional coal or wood burners to process fresh tea leaves) may contribute to the reported contamination (Zhou 2018; Chen and Jiang 2019), while other investigations suggest that the contamination is reduced when these processes are replaced with clean and modern tea production techniques. Although the combustion required by these operations represents an important source of anthraquinone, direct atmospheric contamination by air originating from urban, industrial and biomass burning areas is another likely source of contamination (Romanotto and Gassert 2017; Wang et al. 2018). Plants other than tea, such as currants and peppercorns, could be the subject of similar uptake processes (European Food Safety Authority 2018); however, the affinity of tea leaves for contaminants seems particularly high due to their large surface area, their active metabolism associated with their large number of stomata and the presence of hook-shaped hairs present on the young leaves (Romanotto and Gassert 2017). In this study, we investigate the contamination of tea plantations by anthraquinone from one of the possible sources, atmospheric contamination, and estimate its significance as a source of anthraquinone deposited on fresh tea leaves.

Observations of anthraquinone in the atmosphere are scarce and are based on relatively short time-scale measurements. However, they do cover areas on multiple continents and over different land surfaces, allowing us to gather limited but valuable information about the geographic distribution of this chemical species, its atmospheric concentration levels and variability, and the partitioning between gas and particle phases.

Due to their semi-volatility, PAHs and their oxygenated derivatives (OPAHs), including anthraquinone, exist in the atmosphere in both gas and particulate phases. Typically, most of the anthracene (> 95%), the chemical parent of anthraquinone, is measured in the gas phase, while more than half of the anthraquinone (45–100%) is measured in the particulate phase (Albinet et al. 2008b; Eiguren-Fernandez et al. 2008; Tomaz et al. 2016).

Observations, the oldest one dating back to 1976, show that surface concentrations of anthraquinone are very variable in time and space (see Tables S1, S2 and Fig. S4) with values that usually range from 0.01 to about 100 ng/m^3^. Anthraquinone concentrations are generally higher in urban areas and in areas close to emission sources, such as roads or power plants, suggesting a short atmospheric lifetime for anthraquinone. In the particle phase, it can reach above 100 ng/m^3^ in an urban area (Lin et al. 2015), but it is often several orders of magnitude lower. In remote rural areas (i.e., at Adventdalen near Svalbard), the concentration of anthraquinone falls below 0.2 ng/m^3^ (Drotikova et al. 2020). Anthraquinone is usually considerably more abundant in winter than in summer, particularly in cities (Maria del Rosario and Sienra 2006; Lin et al. 2015). In Beijing, for example, the mean winter concentration (~ 100 ng/m^3^) is two orders of magnitude higher than the mean summer concentration (Lin et al. 2015). A similar ratio is also reported in the city of Grenoble (Tomaz et al. 2017). This seasonal difference is thought to be a result of the higher emission intensity due to high demand of combustion for heating, lower photooxidation rates and more stable boundary layer conditions in winter. In the tropics, the concentrations of anthraquinone are higher during the rain-free season than during and immediately after the monsoon period.

The partitioning between gas and particulate phase of PAHs can significantly affect their atmospheric behavior such as chemical transformation and deposition, and depends on their physical and chemical properties, such as molecular weight and volatility, and on their environmental exposure, such as temperature, humidity and PM concentrations (Lammel et al. 2009). In general, the fraction of these compounds in the particle phase increases with decreasing volatility (Robinson et al. 2007) and therefore with increasing molecular weight and oxygen-to-carbon ratio (Donahue et al. 2009). This gas/particle partitioning also changes seasonally. The fraction of these compounds in particle phase is usually higher in winter than in summer because of the lower temperature that favors condensation. For instance, Albinet et al. (2008a) reported that the fraction of anthraquinone measured in the particulate phase is 95% in winter and 80% in summer.

**Fig. 1:**
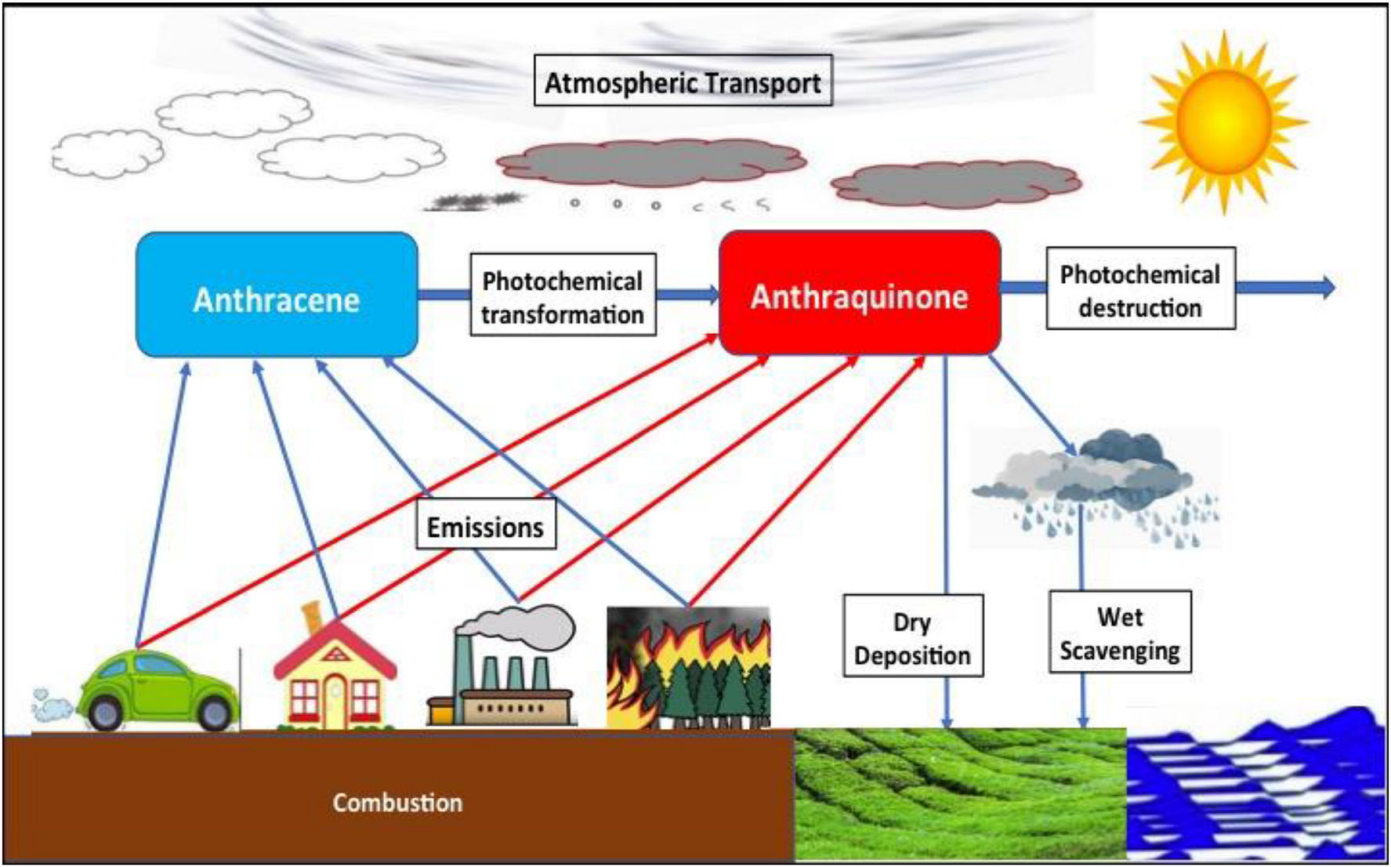
Schematic representation of the key processes affecting the formation and fate of anthraquinone. Major sources and sinks of atmospheric anthraquinone: Anthraquinone is primarily emitted from combustion processes and formed in situ by the oxidation of anthracene. The compound is then destroyed through surface deposition and photochemical destruction

Research on the global atmospheric budget of anthraquinone, including its emissions, the chemical production and destruction, and deposition (Fig. 1), has been limited. Combustion is a major source of this substance, but no reliable surface emission inventory has been developed, so indirect methodologies are required to estimate the emissions. The molar yield of anthraquinone in the oxidation of anthracene by the hydroxyl radical OH is uncertain (Pal and Sharon 2000), but believed to be lower than 50% (Wang et al. 2007; Zeng et al. 2020).

Two possible chemical destruction mechanisms of gas phase and particle phase anthraquinone have been identified in the atmosphere: photo-oxidation by the OH radical and photolysis by solar radiation (International Agency for Research on Cancer 2012; Lin et al. 2015). Anthraquinone is also removed from the atmosphere by wet scavenging in precipitation and dry deposition on the Earth’s surface. The contamination of tea leaves results directly from the uptake of anthraquinone, which is facilitated by the leaf physiology of the plants.

With the existing information on the atmospheric behavior of anthraquinone, we calculate the atmospheric concentration and surface deposition of this OPAH using a global chemical model that accounts for the emissions, global transport, chemical transformations and deposition of anthraquinone in the atmosphere (see “Materials and methods” section). The quantification of these different processes remains uncertain because of the lack of information on key emission and photochemical processes. We quantify and analyze these uncertainties by performing several sensitivity tests based on different assumptions for some of the key model input parameters. Among the wide range of distributions inferred from these sensitivity cases, we select two cases that better represent the gas/particulate partitioning and deposition processes of anthraquinone and present a good match with a wide compilation of atmospheric anthraquinone concentration measurements. These simulations suggest that the deposition of atmospheric anthraquinone could be a substantial source of the anthraquinone content found in tea leaves in several tea-producing regions, especially near highly industrialized and populated areas of southern and eastern Asia.

## Materials and methods

### Model configuration

The transport and fate of anthraquinone in the atmosphere is simulated by the Model for Ozone and Related chemical Tracers, version 4 (MOZART-4), an offline global chemical transport model developed at the National Center for Atmospheric Research (NCAR) (Emmons et al. 2010). For this study, the global model is run for each scenario under consideration during a time period of one year at a spatial horizontal resolution of 0.50° × 0.63°, or approximately 50 km, with 48 vertical levels. Dynamical forcing by meteorological quantities required to simulate long-range transport of chemical species is taken from the GEOS5 Atmospheric Forcing data base for 2013 (Tilmes 2016).

The model is configured to simulate the global distribution of anthracene and anthraquinone for different conditions. The conditions adopted in the simulations discussed in the Main Text (Cases M1-3) are summarized in Table 1. More information on the other simulations (Cases S1-8) can be found in the Supplementary Text and summarized in Table S7. As mentioned above, anthraquinone is emitted primarily from biomass burning, coal burning, and traffic, and is formed secondarily from the photooxidation of anthracene. In order to assess the contribution of different sources to the atmospheric abundance of anthraquinone, specifically in the tea-producing regions, four primarily emitted “tagged” anthraquinone species from each of these main emissions sources are introduced—residential combustion (res), power generation and industry (ene), traffic (tra) and biomass burning (bio). Anthraquinone formation from anthracene photooxidation is accounted for through a fifth tagged anthraquinone species. Since anthracene is mainly emitted from sources similar to those of anthraquinone, a similar tagging is applied for anthracene.

**Table 1:** Summary of Model Scenarios. Summary of the different simulation cases (M1-3) mentioned in the Main Text, with different assumptions for the emission scenario (E2 & E3), for the yield of anthraquinone formation from anthracene photo-oxidation (Y1) and for the anthraquinone destruction rate (D1 & D2). The summary of all simulation cases (M1-3 & S1-8) adopted in the present study is provided in Table S7

Simulation	Description of case	Emission scenario	AQ production yield	AQ destruction time scale (overhead Sun)
M1	High chemical production, low loss, highest seasonality in emissions	Enhanced seasonality + enhanced winter emission (E3)	50% (Y1)	10 days (D1)
M2	High chemical production, highest loss, high seasonality in emissions	Enhanced seasonality relative to PKU-PAH inventory (E2)	50% (Y1)	20 min (D2)
M3	High chemical production, low loss, reduced residential emissions from M2 high seasonality in emissions	Residential emissions of E2 reduced by 66%	50% (Y1)	10 days (D1)

### Surface emissions of anthracene and anthraquinone

In the absence of emission inventories for anthraquinone, we base our estimate on the emission of its parent species, anthracene, for which a global inventory is available for 2014 from Peking University (Shen et al. 2013) (referred to as the PKU-PAH inventory) for year 2014 at a spatial resolution to 0.1° × 0.1° (about 10 × 10 km^2^) and a temporal resolution of one month^2^. The 6 economic sectors of the inventory are mapped into the 4 emission categories of the tagged model species (res, ene, tra and bio, see above). The mapping of the sectors in the PKU-PAH sectors to the anthracene species in our model is summarized in Table S4.

We make the assumption that the emission of anthraquinone is proportional to the emission of anthracene with proportionality factors provided by the ratio between the emission factors of anthracene (EF_ANT_) and anthraquinone (EF_AQ_) for which some information is available. The mapping of the surface emissions of anthracene and anthraquinone based on the PKU-PAH inventory and referred to as scenario E1 is shown in Table S4.

Measurements suggest that the concentration ratio for anthraquinone between winter and summer can reach a factor 10 to 100 (Lin et al. 2015), which is considerably higher than the emission ratio derived from the PKU-PAH inventory. Since emissions appear to be particularly sensitive to the high fuel consumption during the winter season (Zhang and Tao 2008), we consider a second scenario (called here emission scenario E2) in which the seasonal variation of the anthraquinone emissions for the residential sector is scaled to the monthly profiles of carbon monoxide emissions as provided by Guevara et al. (Guevara et al. 2020).

Coal consumption and related emissions could be underestimated in northern China and northern India (Cheng et al. 2017). As result, we are considering a third scenario (called emission scenario E3) in which the emissions of anthraquinone in the residential sector are enhanced by a factor 2.5 in Northern China and Northern India during the heating months between November and February. The regions experiencing this enhancement are shown in Fig. S3. We adopt this emission scenario in our baseline simulation M1.

Re-volatilization of anthracene and anthraquinone is not considered in this study as its role is negligible compared to direct emissions (see Supplementary Text for a more elaborate discussion).

### Chemical production and destruction of anthraquinone

Both anthraquinone and anthracene are semi-volatile organic compounds that can be present in the atmosphere in gaseous and particulate phase. The partitioning between the gaseous and particulate phase depends on ambient atmospheric conditions such as temperature and aerosol concentration. The mass fraction in the particulate phase is given by $\theta = c_{p} /(c_{p} + c_{g} )$, where *c*_*p*_ and *c*_*g*_ are the concentrations of the compound in the particulate and gaseous phase respectively (both in units of μg m^−3^). Their partitioning between the two phases is quantified by the gas-particle partitioning coefficient *K*_*p*_ (m^3^ μg^−1^): $K_{p} = c_{p} /(c_{g} \cdot M)$, where *M* (μg m^−3^) is the aerosol mass concentration. When the gas/particulate partitioning is dominated by absorption onto aerosol surface, *K*_*p*_ can be expressed as a formulation dependent on the temperature *T* (K) (Takekawa et al. 2003): $K_{p} = K_{p,298} \cdot \frac{T}{298} \cdot \exp \left[ {\frac{{Q_{v} }}{R}\left( {\frac{1}{T} - \frac{1}{298}} \right)} \right]{ },$ where *Q*_*v*_ is the enthalpy of vaporization of the sub-cooled (super-cooled) liquid, *R* is the gas constant and *K*_*p*,298_ is the value of *K*_*p*_ at 298 K. The corresponding values *K*_*p*,298_ and *Q*_*v*_ of anthraquinone and anthracene are derived by regression of in situ particulate mass fraction measurements (see Supplementary Text for detailed description). The gas/particulate partitioning of anthraquinone affects its dry and wet deposition.

Apart from direct surface emissions, anthraquinone is produced from the photo-oxidation of anthracene (ANT), initiated by the hydroxyl radical OH (ANT + OH → AQ) (Goulay et al. 2005; Manion et al. 2008; McGillen et al. 2020). This reaction is a chain reaction (Pal and Sharon 2000), and the production yield of anthraquinone from this reaction is unknown. The yield adopted in our model is based on existing information on chemical processes affecting similar molecules (see Supplement). An upper limit of the production yield of 50% is adopted in the production scenario Y1 (Zeng et al. 2020), and a lower limit of 10% (Wang et al. 2007) in the production scenario Y2 (see Table 1). The large uncertainty on the chemical formation of anthraquinone is expected to have limited effects on the calculated atmospheric concentrations because the contribution of direct emissions usually dominates.

For the destruction of anthraquinone in the atmosphere, we consider several possible mechanisms. One of them is through the photo-oxidation by the OH radical (Scenario D1). The time scale for anthraquinone destruction by this reaction is around 10 days (Wang et al. 2007). The second destruction mechanism is photolysis. For this particular process, we consider three scenarios with different photolysis time constant at overhead Sun and at all altitudes of 20 min (Scenario D2), 40 min (Scenario D3) and 1 h (Scenario D4), respectively. These lifetimes are chosen according to the photolysis lifetime of other similar chemical compounds obtained from existing measurements (Atkinson et al. 1989; U.S. National Library of Medicine 2006). Although the destruction lifetime of anthraquinone is uncertain, its precise value is of relatively little importance for the estimation of AQ deposition, as to be shown in the Results section.

### Dry and wet deposition of anthracene and anthraquinone

Dry deposition represents a major sink for anthracene and anthraquinone, and is parameterized in terms of a deposition velocity that varies according to land surfaces. Over the continent, we assume that anthracene is deposited with an effective velocity of 0.24 cm s^−1^ (Chang et al. 2003). Over water surfaces, we imposed a lower deposition velocity of 0.05 cm s^−1^, as the solubility of anthracene is low (Yalkowsky et al. 2016).

The deposition velocity for gaseous-phase anthraquinone is a function of the mass fraction in particulate phase of anthraquinone and the fraction of land use categories in a grid. With the information on the uptake of anthraquinone being very limited, we derive a value for the deposition velocity based on measurements made for other gas-phase organic compounds with similar chemical properties. As detailed in the Supplement, we estimate the deposition velocity of gas-phase anthraquinone to be 0.7 cm s^−1^ over vegetated land. For anthraquinone in the particle phase, the deposition velocity depends highly on the size of the particles on which anthraquinone condenses. We therefore estimate the deposition velocity to be 0.15 cm s^−1^ for anthraquinone in the particle phase over vegetated land.

As anthracene resides mostly in the gaseous phase and its solubility in water is low, the wet scavenging for anthracene is neglected in all of the simulations. For anthraquinone, the removal by rainout is dependent on the temperature-dependent particulate mass fraction. The loss rate for gaseous anthraquinone is a function of the rainwater tendency, the mass density of convective and non-convective raindrops and ambient temperature (Giorgi and Chameides 1985). For particulate anthraquinone, the loss rate is set to 20% of the loss rate of nitric acid (Emmons et al. 2010).

## Results

### Global distributions and seasonal variations of anthraquinone emissions

**Fig. 2:**
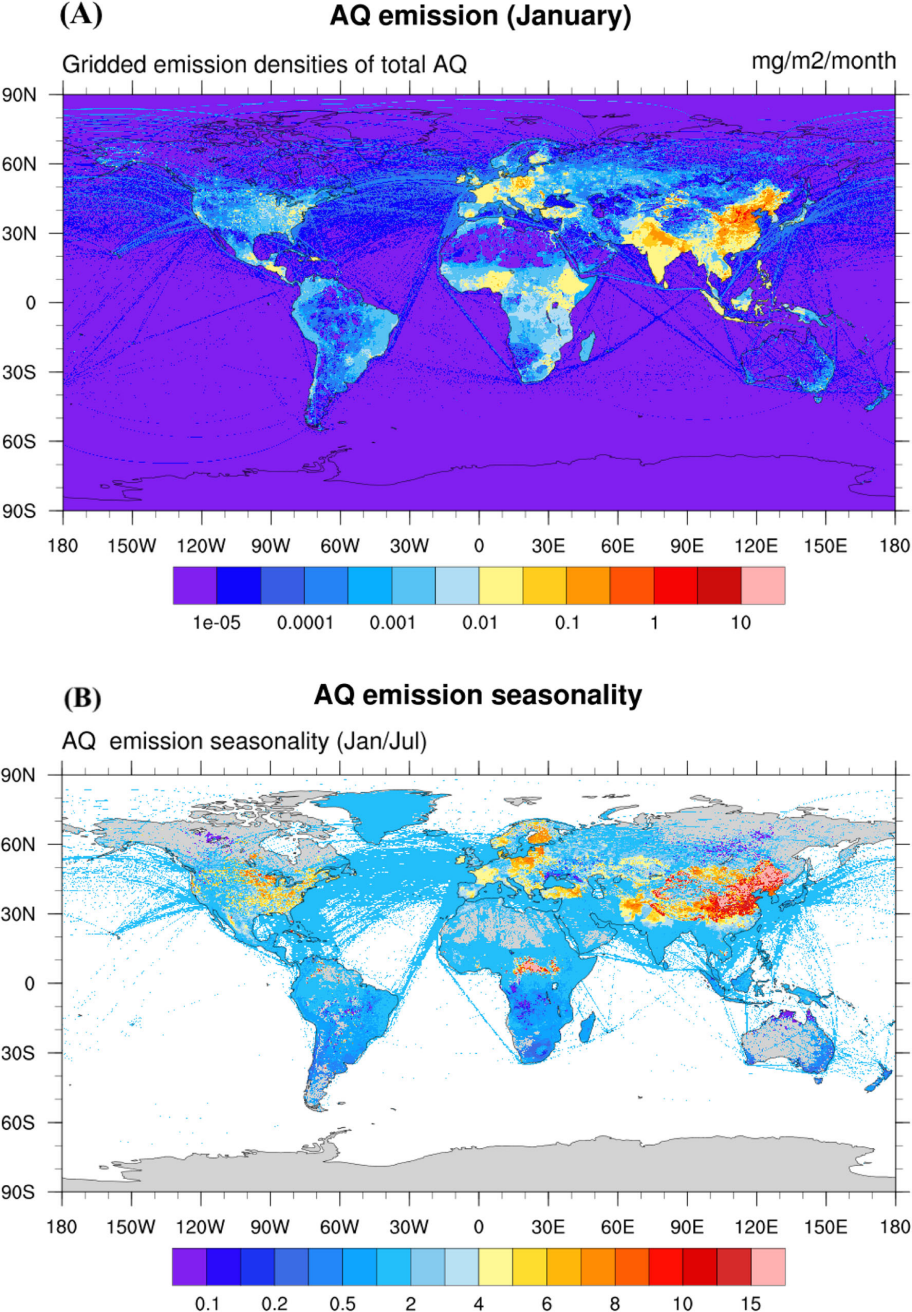
Global distribution of AQ surface emissions. **A** Global distribution of the estimated surface emissions of anthraquinone (molecules cm^−2^ s^−1^) in January (top panel) and **B** their seasonality (ratio between January and July mean values). These refer to the emission scenario E3 (see Table 1)

Figure 2A shows the AQ emissions for the month of January and Fig. 2B the ratio between January and July values (both for emission scenario E3). We notice the existence of emission hotspots in Northeastern China, Northern India and Eastern Europe. High emissions are also found near large urban areas, particularly in East Asia, Europe and in the US with, in addition, some minor hotspots in Indonesia, parts of Africa (e. g. Nigeria and Ethiopia) and Central America. In the latter regions, the emissions are attributed to forest fires associated with agricultural practices and deforestation. In the adopted case, a factor of about 10 is derived for the winter-to-summer emission ratios in the most populated areas of both hemispheres at latitudes above 30°. The seasonality is noticeably weaker in Western Europe and in the West Coast of the US due to the less pronounced seasonal variation in temperature (Guevara et al. 2020). This ratio, however, is reduced to a factor of 3–4 in those areas if we adopt the PKU-PAH emission inventory (Shen et al. 2013) (scenario E1) without correction.

With our adopted assumptions (Scenario E3), the global annual emission of anthraquinone is estimated to be close to 11 Gg/yr. It is 18% lower, however, if our correction on the winter residential emissions is not applied (Scenario E1 and E2). For all three scenarios, residential combustion is the largest source of anthraquinone. In Scenario E3, it contributes 83.7% of the global annual total emissions, followed by biomass burning at 6.4%, traffic at 6.0%, and energy generation and industry at 3.9%.

**Fig. 3:**
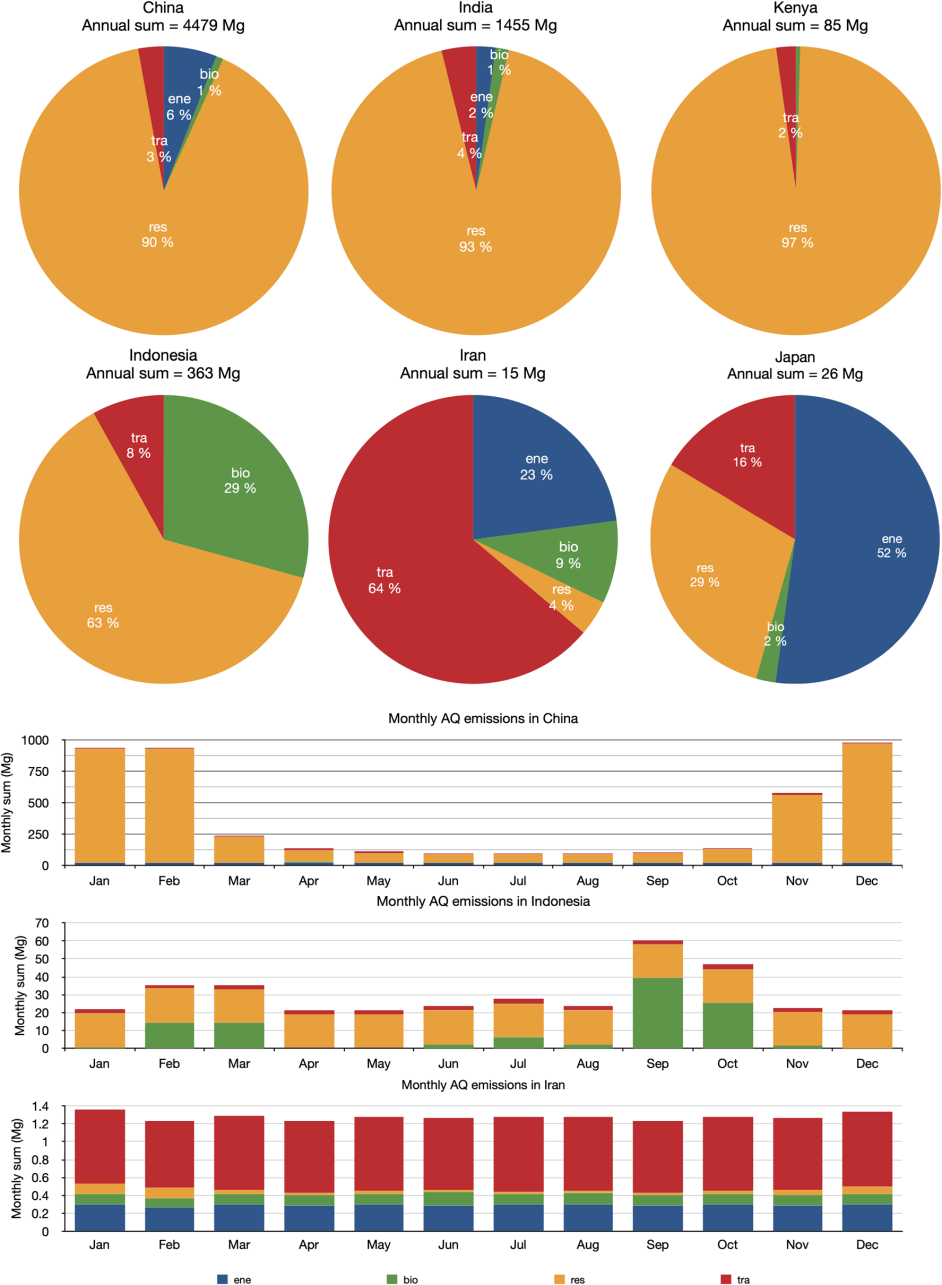
Sectoral contributions of anthraquinone emissions in key tea-producing countries. Sectoral contribution of anthraquinone emissions in six tea-producing countries (top panel) and the seasonal variation of the emission per sector in China, Indonesia and Iran (bottom panel) derived for the emission scenario E3. The sectors are energy production (ene), biomass burning (bio), residential (res), and transport (tra)

The two top tea-producing countries are China, which emits 4.48 Gg of anthraquinone per year or 3.11 g/year per capita, and India, which emits a corresponding amount of 1.46 Gg/year, or 1.05 g/year per capita. Emissions from residential combustion are the dominant source of anthraquinone in the top 3 tea-producing countries, i.e., China (90%), India (93%) and Kenya (97%) (see Fig. 3 top panel). China and India follow similar seasonal variations with substantially larger emissions from the residential sector in winter (see Fig. 3 bottom panel). For example, monthly averaged anthraquinone emissions from the residential sector during the heating season (November to March; 703 Mg) are ~ 9 times higher than in the non-heating season (April to October; 77 Mg) in China. Countries experiencing less seasonal variations in temperature, such as Kenya and Vietnam, have a relatively constant contribution from the residential sector throughout the year (see Fig. 3 bottom panel). In other countries such as Indonesia, a major source is associated with biomass burning with pronounced peaks during the seasons of deforestation/wildfire (February to Mar and June to August) and agricultural activities (September to October). In Iran, more than half of the anthraquinone is emitted from the traffic sector with little seasonality. The dominant contribution in high-income countries, such as Japan, is from the power generation and industry sectors with a relatively low contribution from the residential sector due to the insignificant contribution from crop and wood burning in the residential sector.

As, in addition to the uncertainties in the emissions, in situ chemical production and loss rates of anthraquinone are poorly quantified, we consider several sensitivity cases (Cases S1-8) with our global simulation of atmospheric anthraquinone as described in Table S7. In addition to the different assumptions made for the emissions, we assume two different yields (50% in scenario Y1 and 10% in scenario Y2) for the photochemical AQ production from the photooxidation of anthracene, and four different formulations (D1-4) for the atmospheric destruction of anthraquinone, as described in the Section in “Materials and Methods” and Supplementary Text. With the comparison between the model results from these sensitivity cases and atmospheric observations, the simulations with AQ emissions with enhanced seasonality (Scenario E2 or E3) and the 50% AQ production yield (Scenario Y1) provide better agreement with the measurements. Therefore, two simulations (Cases M1 & M2) are conducted with these scenarios with the implementation of a more detailed treatment of anthraquinone chemistry and deposition that account for temperature-dependent gas/particulate partitioning of anthraquinone. Case M1 (E3 emission scenario, 50% AQ yield (Y1) and a very slow AQ chemical loss (D1)) provides the best agreement with observational data (see Supplementary Text), and is therefore adopted as our baseline case. Case M2, which assumes very fast AQ photochemical loss (D2) resulting in lower AQ concentrations, will be also considered. Case M3 is a mitigation scenario. The model settings of Case M1-3 are listed in Table 1, with details in the Section on “Materials and Methods”.

### Surface concentrations of anthraquinone

**Fig. 4:**
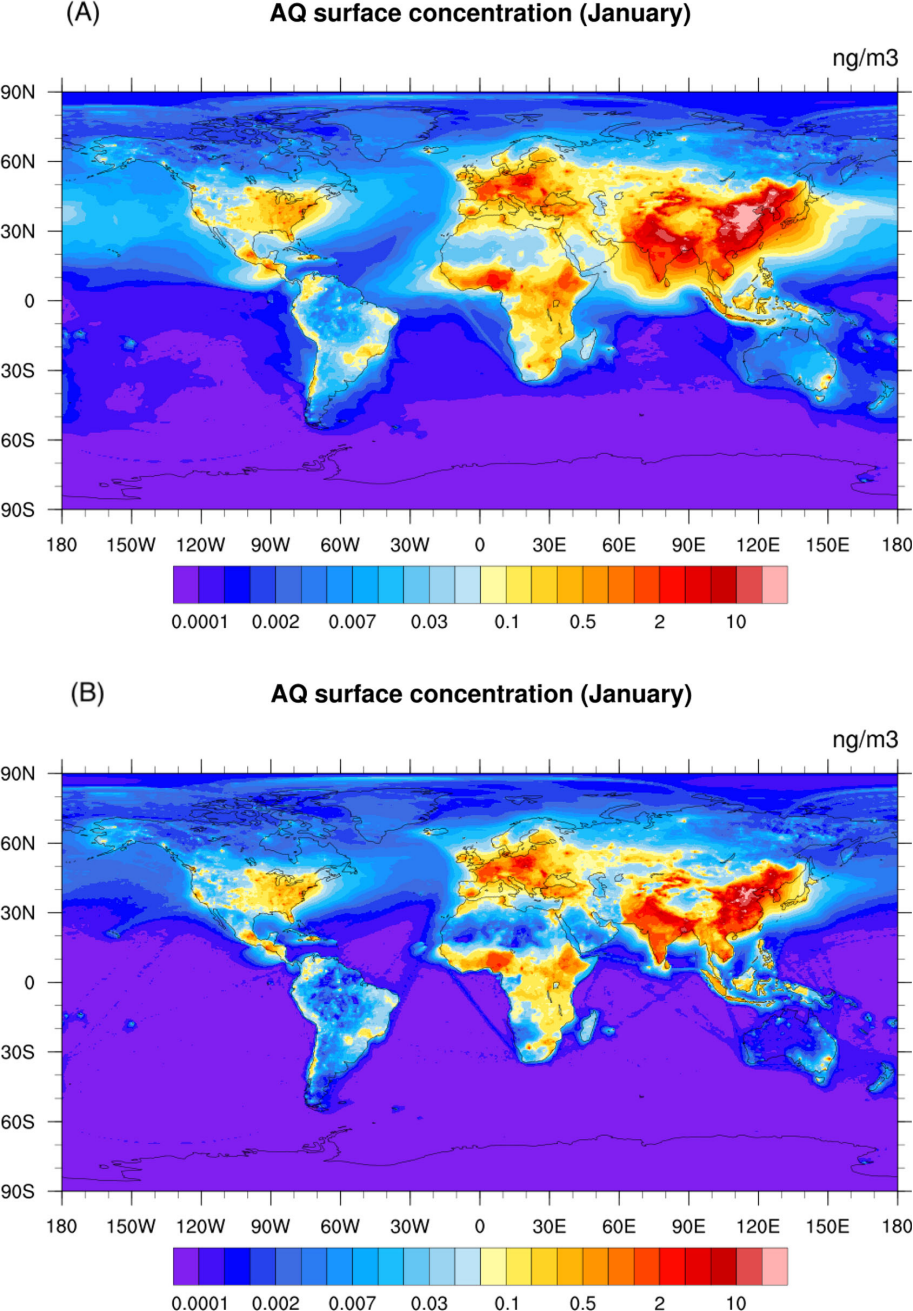

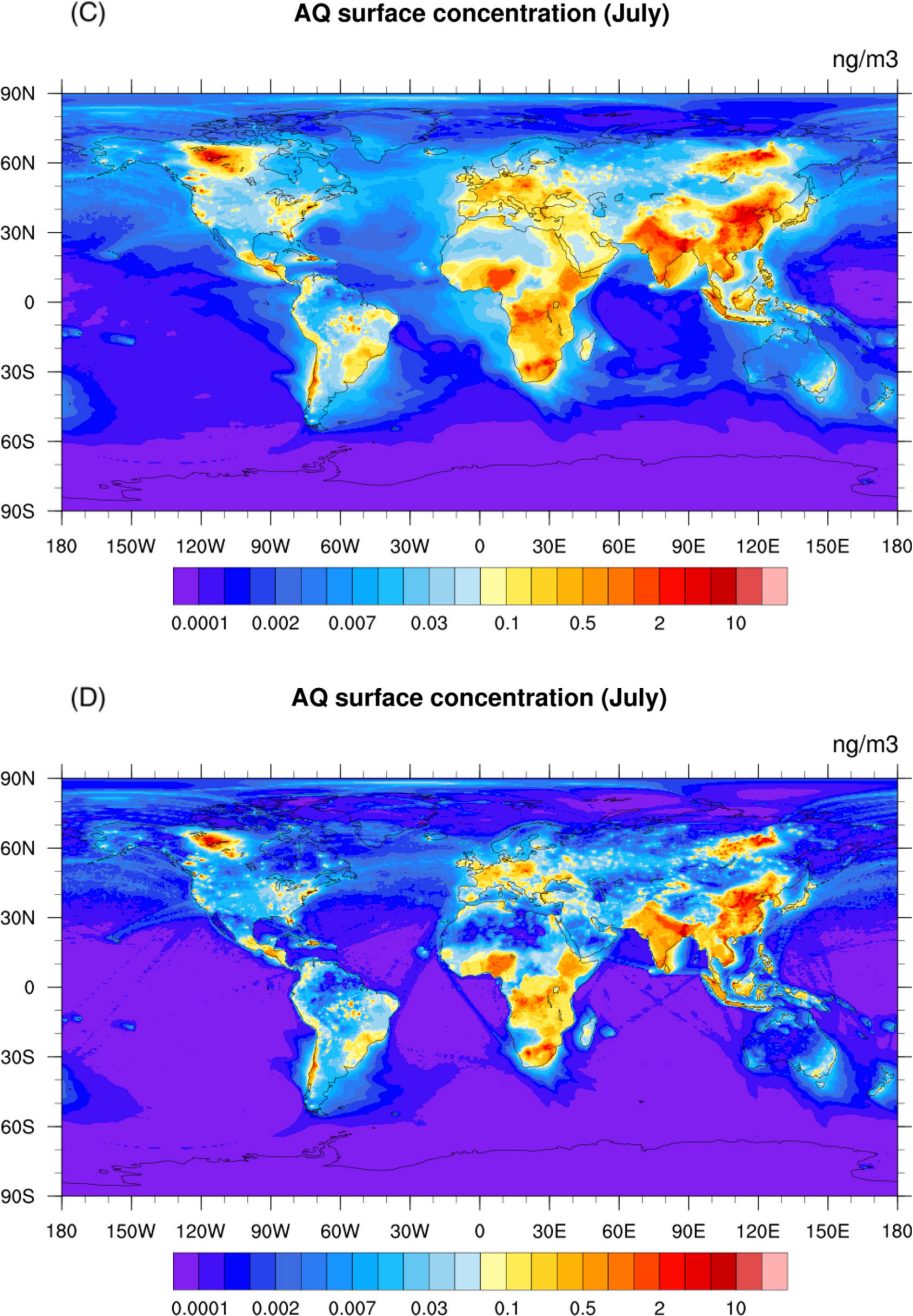
Global distribution of AQ surface concentrations. Global distribution of the January (Panel **A**, **B**) and July (Panel **C**, **D**) surface concentration (ng/m^3^) of anthraquinone calculated for two model assumptions: Panel **A** and **C** (Case M1): with higher winter emissions (E3), 50% AQ yield (Y1) and 10-day AQ photochemical loss (D1); and Panel **B** and **D** (Case M2): with normal winter emissions (E2), 50% AQ yield (Y1) and 20-min AQ photolysis loss (D2)

With this estimation of the emission, production and destruction rates and the constraints provided by atmospheric observations, we derive the global atmospheric distribution of the surface concentration of anthraquinone (Fig. 4) from different model simulations (Cases M1 and M2, see Table 1) using a global chemical-transport model of the atmosphere (Emmons et al. 2010). In all model cases, the largest surface mixing ratios are derived in the tea-producing countries of Asia. In January, the concentrations calculated in the baseline case M1 (Fig. 4A) reach 20 ng/m^3^ in northern China and in the Ganges River Valley of India. High levels of anthraquinone are also noticeable in Eastern Europe, particularly Poland (3 ng/m^3^) due to intense coal combustion and, to a lesser extent, in parts of Africa (~ 1 ng/m^3^) where residential cooking is a significant source. With the adopted model assumptions, the concentration patterns closely follow the geographic distribution of the surface emissions, although plumes extending over the neighboring oceans are noticed. However, if one adopts a higher loss rate (Case M2 with destruction characteristic time of 20 min for overhead Sun (D4); see Fig. 4B), the calculated concentrations in the hot spots of Asia, Europe and Africa are similar as in the previous case, but the global distribution is characterized by less spatial dispersion over the continents, in particular over oceans. In fact, with a lifetime of 10 days (Case M1), the influences of the anthraquinone emissions are far-reaching, while they are considerably more localized when we adopt a shorter lifetime as in Case M2.

**Fig. 5:**
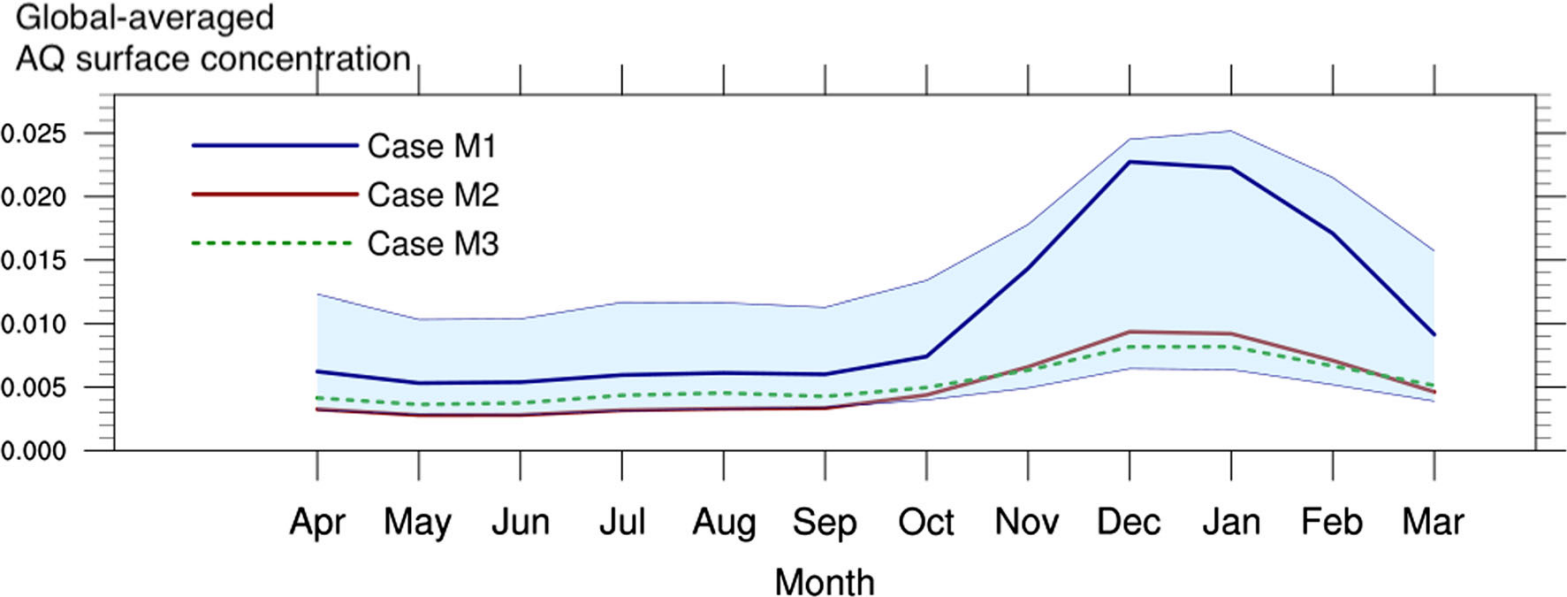
Global average AQ total concentration. Annual evolution of the global average AQ total concentration from different cases: *blue solid*: Case M1, baseline case (highest winter emissions (E3), 50% AQ yield (Y1), 10-day chemical lifetime (D1); *red solid*: Case M2 (with E2 emissions), 50% AQ yield (Y1) and 20-min photolysis lifetime for overhead Sun (D2)); *green dotted*: Case M3, same as Case M1 but with reduced residential emissions

The model shows reasonable agreement with the observational data, as detailed in Supplementary Text and Table S8. The best performance against data at background sites is realized by the baseline run (Case M1) which achieves the highest correlation (Pearson correlation coefficient *r* = 0.69) and lowest bias (normalized mean bias *NMB* = − 7%). The other run (Case M2) also achieves good correlation (0.65) but underestimates the background data by almost 50%. Both simulations M1 & M2 underestimate the non-background observations, by 8% and 54%, respectively. Underestimation is expected, however, due to the relatively coarse resolution of the model (~ 50 km) compared to urban scales (~ 10 km), which may dilute the emission hotspots located in/near cities. Figure 5 shows the global average surface concentration of anthraquinone throughout the year provided by Case M1 & M2.

With the baseline case M1, we find that the largest contribution to the global annual-mean surface concentration of anthraquinone is provided by the residential sector (65%). The second largest contribution results from the secondary formation by the oxidation of anthracene (24%), followed by the contribution from traffic and biomass burning (4% for both), and power generation and industry (3%). Residential combustion and biomass burning exhibit strong seasonal variations. For example, the contribution from residential combustion is substantially larger in the northern hemisphere winter (October to March; 73%) than in the northern hemisphere summer (April to September; 44%) due to high demand in residential heating during the cold period. Biomass burning, on the contrary, contributes more in the northern hemisphere summer (10%) than in the winter (2%). The other sectors show relatively constant contributions throughout the year.

## Discussion

### Surface deposition of anthraquinone and deposition onto tea leaves

**Fig. 6:**
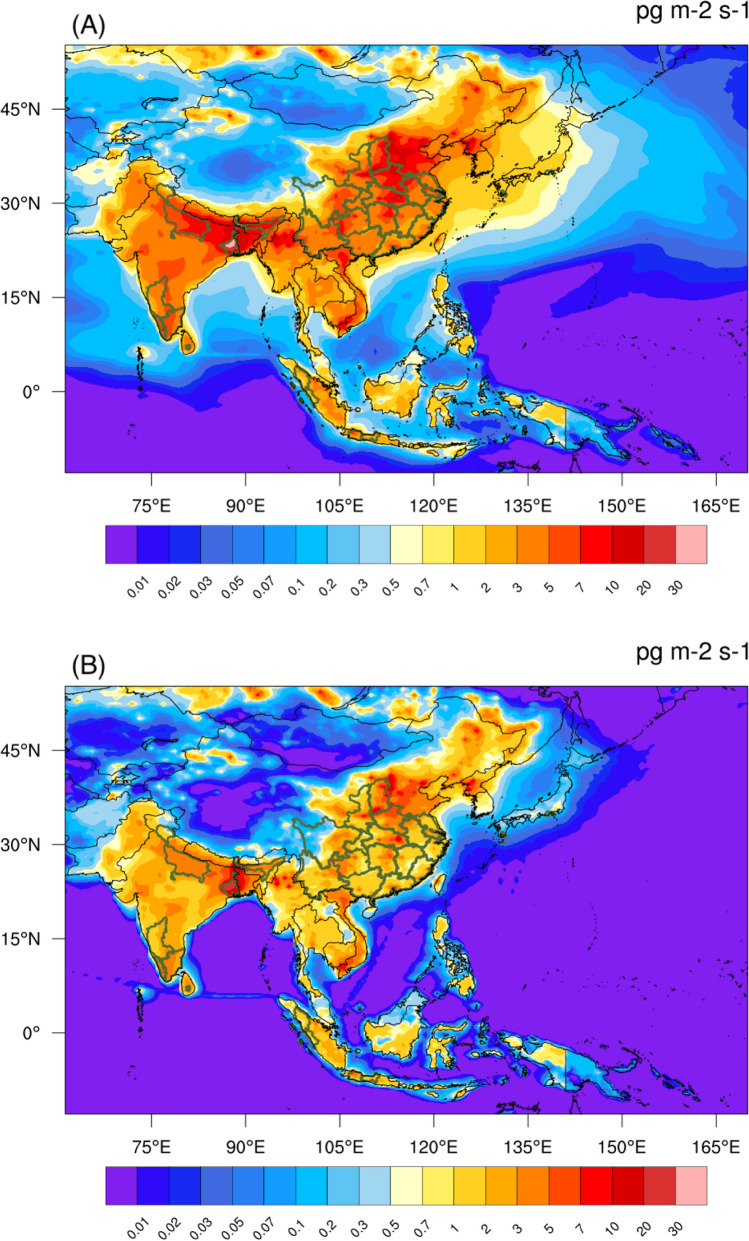
Distribution of AQ deposition in Asia. Calculated distribution of anthraquinone deposition rate (in pg m^−2^ s^−1^) in east and south Asia during the month of April for simulation case M1 (Panel (**A**)) and case M2 (Panel (**B**)). The key tea producing provinces of the countries in east and south Asia are marked by the green border (tea producing map provided by private communications with members of the Intergovernmental Group (IGG) on Tea of the Food and Agriculture Organization of the United Nations (FAO)). The larger geographical spread seen in the field derived in Case M1 is due to the longer chemical lifetime adopted in this particular scenario

The rate of surface deposition flux of anthraquinone is particularly high in southern and eastern Asia, where the surface concentration values derived from the model are large. The values depend not only on the adopted deposition velocities (See “Materials and methods” section), but also on the assumptions made for the surface emissions and for the atmospheric loss rates. We display in Fig. 6 two specific estimates of the deposition rates in east and south Asia (Cases M1 and M2) during the month of April. The key tea-producing regions are indicated with green borders. The simulation adopting the longer chemical destruction time (Case M1) leads to higher deposition and hence potentially to higher contamination of tea leaves. In both cases, some of the locations with high deposition of anthraquinone coincide with certain key tea-producing regions. The simulations suggest that the highest deposition rate occurs in the northern parts of China and India. Among the key tea-producing regions, the province of Hubei in China and the province of West Bengal in India are those where tea plantations are expected to be exposed to the highest levels of anthraquinone deposition. The corresponding deposition is found to be smallest during the summer months.

The estimated amount of anthraquinone deposited on tea leaves is derived by integrating the deposition rate of this molecule during the exposure time of the leaf. The calculation must account for the average number of leaves per unit area of the tea plantation (Carr 2018; Wang et al. 2020) and the plucking frequency (4–16 days) of the tea leaves (Tripathi et al. 2004; Sylvestre et al. 2014; Hajiboland 2017), as well as the possible loss of the deposit due, for example, to photolysis and chemical reactions. For a deposition rate of 10 pg m^−2^ s^−1^ (Fig. 6), accumulating over a period of 10 days, the amount of anthraquinone deposited on a tea-planted area during this period would be equal to 8.6 μg m^−2^. With 350 shoots per m^2^ and 3 leaves per shoot in a typical tea farm (Carr 2018), this corresponds to a maximum of 8.2 ng per leaf, which is equivalent to 0.033 mg per kg of fresh leaves, with a typical fresh tea leaf weighing 0.25 g (Carr 2018).

The calculation of the anthraquinone content contained in the dry and processed tea leaves must also account for the impact of the AQ dissipation from tea shoots and the successive manufacturing steps leading to the final product. For instance, the laboratory experiment by Wang et al. (2018) reported the half-life of the decay of the anthraquinone residue on tea leaves due to dissipation is 3.7 days (which is equivalent to an exponential decay timescale of 5.34 days). This results in 27% decrease in the AQ residue from the deposited amount until the tea leaves are harvested. A further 58.8–84.6% of AQ residues are lost during the tea processing steps (including withering, de-enzyme, fermentation and drying for black tea, and de-enzyme, rolling and drying for green tea) (Wang et al. 2018). With a typical moisture content ranging from 350 to 400% on a dry mass basis (Zhang and Okamura 2000), the expected AQ residue contributed by atmospheric contamination calculated here could exceed the MRL set by the European Union (currently set at 0.02 mg per kg of dry tea levels) (The European Parliament and the Council of the European Union 2005). Additional contamination could also result from other local effects associated with tea production operations.

### Mitigation

Finally, we examine the effect of a mitigation scenario in which the emissions of anthraquinone are substantially reduced. In addition to measures to be taken to improve the tea production processes, actions could be undertaken to reduce combustion in the larger regions surrounding tea plantations, particularly in the residential sector. For example, it is estimated that, if all traditional wood and coal stoves used for cooking and heating were replaced by modern systems, up to 50% of the corresponding anthraquinone emissions would be eliminated. A switch to cleaner fuels for residential heating would also be beneficial. To test the effect of this type of mitigation strategy, we conduct an additional simulation (Case M3) in which the level of anthraquinone emissions from the residential sector, which contributes most to near-surface concentrations of this substance, is reduced by two-thirds (relative to simulation case M1 in Table 1).

Under this scenario, the near-surface concentration of anthraquinone is reduced by 30–90% in most of the high-concentration regions (see Fig. S1), and specifically, the annual-mean values in China and India are reduced by more than a factor of two (from 2.66 to 0.99 ng/m^3^, and from 2.20 to 1.04 ng/m^3^, respectively). The resulting global average surface concentration of anthraquinone throughout the year is shown in Fig. 5. Although a decrease in residential emissions is not expected to fully eliminate anthraquinone contamination of tea leaves, it would reduce the size of the high-risk contamination areas and narrow the time window of the contamination.

## Conclusions

In summary, in addition to the direct sources of anthraquinone generated during tea manufacturing, atmospheric deposition of this organic species emitted by combustion from residential and industrial activities as well as from biomass burning constitutes a potentially significant source of contamination of tea leaves. Uncertainties in the surface emissions of anthraquinone and on parameters that determine the atmospheric formation and destruction of this oxygenated polyaromatic hydrocarbon do not allow us to provide definitive quantitative conclusions. Our study, however, shows that for certain values of the adopted parameters, the anthraquinone deposition on tea leaves could exceed the Maximum Residue Limit defined by the European Union. It is worth noting that among the different model simulations, the highest modeled AQ deposition rates are realized by the scenario (M1) which agrees most closely with atmospheric AQ observations.

A more detailed and definitive assessment of the potential contamination of tea leaves requires more quantitative experimental investigations on the atmospheric abundance and the surface fluxes of this substance in different environments as well as comprehensive laboratory studies of the photochemical processes affecting the formation and fate of this molecule. With the limited present state of knowledge on the physical and chemical parameters affecting anthraquinone, we can only conclude that there is a strong possibility tea leaves contamination resulting from residential and traffic sources exceeds the MRL limit with potential health risks for tea consumers. Additional contamination probably results from local tea production processes. More investigations on the impact of the manufacturing process on the anthraquinone content in tea leaves, such as those conducted by Wang et al. (2018), will therefore be essential to provide a comprehensive assessment on the source of anthraquinone residue found in the final tea products. In the short-term, the development of economically feasible mitigation strategies including the conversion of old wood and coal stoves into environmentally friendly facilities should be beneficial.

## Supplementary Information

Below is the link to the electronic supplementary material.

Supplementary file1 (PDF 1658 KB)

## Footnotes

1 This MRL of anthraquinone is set at the lower limit of analytical determination.

2 http://inventory.pku.edu.cn/download/download.html, last accessed on 16 March 2023.
