# Supplementary material for: Contamination of tea leaves by anthraquinone: The atmosphere as a possible source
Source: Ambio. 2023 Apr 28;52(8):1373–88. doi: 10.1007/s13280-023-01858-9 (PMC10272057; doi:10.1007/s13280-023-01858-9)
Supplement: Supplementary file 1 — Supplementary file1 (PDF 1658 KB) [file 13280_2023_1858_MOESM1_ESM.pdf]

Ambio

Supplementary Information
This supplementary information has not been peer reviewed.

Title: **Contamination of tea leaves by anthraquinone: The atmosphere as a possible source**

Authors: Cathy W. Y. Li, Stacy Walters, Jean-François Müller, John Orlando and Guy P. Brasseur

Supplementary Information Text

Additional Information on Materials and Methods

Model description

The transport and fate of anthraquinone in the atmosphere is simulated by the Model for Ozone and Related chemical Tracers, version 4 (MOZART-4), an offline global chemical transport model developed at the National Center for Atmospheric Research (NCAR) (Emmons et al., 2010). For this study, the global model is run for each scenario under consideration during a time period of one year at a spatial horizontal resolution of 0.50° × 0.63°, or approximately 50 km, with 48 vertical levels. Dynamical forcing by meteorological quantities required to simulate long-range transport of chemical species is taken from the GEOS5 Atmospheric Forcing data base for 2013 (Tilmes, 2016).

The model is configured to simulate the global distribution of anthracene and anthraquinone for different conditions. The conditions adopted the simulations discussed in the Main Text (Cases M1-3) are summarized in Table 1. More description of the other simulations (Cases S1-8) can be found in the Supplementary Text and summarized in Table S7. As mentioned above, anthraquinone is emitted primarily from biomass burning, coal burning, and traffic, and is formed secondarily from the photooxidation of anthracene. To assess the contribution of different sources to the atmospheric abundance of anthraquinone, specifically in the tea-producing regions, four primarily-emitted "tagged" anthraquinone species from each of these main emissions sources are introduced - residential combustion (res), power generation and industry (ene), traffic (tra) and biomass burning (bio). Anthraquinone formation from anthracene oxidation is accounted for through a fifth tagged anthraquinone species. Since anthracene is mainly emitted from sources similar to those of anthraquinone, a similar tagging is applied for anthracene.

Surface emissions of anthracene and anthraquinone

In the absence of emission inventories for anthraquinone, we base our estimate on the emission of its parent species, anthracene, for which a global inventory is available for 2014 from Peking University (Shen et al., 2013) (referred to as the PKU-PAH inventory) for year 2014 at a spatial resolution to 0.1° × 0.1° (about 10 × 10 km^2) and a temporal resolution of one month (http://inventory.pku.edu.cn/download/download.html, last accessed on 16 March 2023). We combine the 6 economic sectors of the inventory into the 4 emission categories of the tagged model species (res, ene, tra and bio, see above). The mapping of the sectors in the PKU-PAH sectors to the anthracene species in our model is summarized in Table S4.

We make the assumption that the emission of anthraquinone is proportional to the emission of anthracene with proportionality factors provided by the ratio between the emission factors of anthracene (EF_{ANT}) and anthraquinone (EF_{AQ}) for which some information is available. In the case of road traffic (res), we adopt for the ratio EF_{AQ} /EF_{ANT} the values of 0.50 and 0.65 for gasoline and diesel vehicles respectively, based on Zielinska et al. (Zielinska et al., 2004) (EF_{ANT} = 68.13 and 11.01 micrograms/mile and EF_{AQ} = 34.15 and 7.15 micrograms/mile in the case of gasoline and diesel vehicles, respectively). We represent the overall emission of road traffic by averaging the contribution of gasoline and diesel vehicles.

The adopted emission factors for the residential combustion sector (res; e.g., heating, cooking) are based on the measurements made by Shen (Shen, 2014) for 5 types of coal, 9 types of crops and 27 types of wood. These factors are obtained by averaging the contribution of the different types of fuels with resultant values being equal to 0.26, 0.67 and 0.82 kg anthraquinone per kg of anthracene emitted for coal, crop and wood burning, respectively. Based on the PKU-PAH/PKU-FUEL inventory (Wang et al., 2013), the fuel consumption in the residential sector is dominated by biomass burning, in particular crop (26%) and wood burning (23%), while coal represents only 3% . The resulting emission factors for residential heating are derived as the average between the contributions of crop and wood burning. Information regarding these emission factors is provided

in Table S3. Since no information is available for the sectors of energy production and industry (ene), we adopt the emission factor ratio derived for coal burning since a large number of power generation facilities belong to this particular sector. For the biomass burning sector (bio), we adopt the average emission ratio for crop and wood burning. The mapping of the surface emissions of anthracene and anthraquinone based on the PKU-PAH inventory and referred to as scenario E1 is shown in Table S4.

Measurements suggest that the AQ concentration ratio between winter and summer can reach a factor 10 to 100, which is considerably higher than the emission ratio derived from the PKU-PAH inventory. Since emissions appear to be particularly sensitive to the high fuel consumption during the winter season (Zhang and Tao, 2008), we consider a second scenario (called here emission scenario E2) in which the seasonal variation of the anthraquinone emissions for the residential sector is scaled to the monthly profiles of carbon monoxide emissions as provided by Guevara et al. (Guevara et al., 2020). This approach is based on the high correlation existing between the emissions of CO and the emissions of anthracene (see Figure S2).

According to Cheng et al. (Cheng et al., 2017), the actual consumption in 2013 of residential coal in the Beijing-Tianjin-Hebei (BTH) region is considerably higher than the consumption reported by the 2014 China Energy Statistical Yearbook (0.7 versus 0.15 tons per year). When the higher estimates are used, the emissions due to fossil fuel combustion are about a factor 2.5 higher (see Table S5). Coal consumption and related emissions could also be underestimated in India. As result, we are considering a third scenario (called emission scenario E3) in which the emissions of anthraquinone in the residential sector are enhanced by a factor 2.5 in Northern China and Northern India during the heating months between November and February. The regions experiencing this enhancement are shown in Figure S3. We adopt this emission scenario in our baseline simulation M1.

Temperature-dependent gas/particulate partitioning

Both anthraquinone and anthracene are semi-volatile organic compounds that can be present in the atmosphere in gaseous and particulate phase. The partitioning between the gaseous and particulate phase depends on ambient atmospheric conditions such as temperature and aerosol concentration. The mass fraction in the particulate phase is given by $\theta = c_p/(c_p + c_g)$, where c_p and c_g are the concentrations of the compound in the particulate and gaseous phase respectively (both in units of μg m^{-3}). Their partitioning between the two phases can be quantified by the gas-particle partitioning coefficient K_p (m^3 μg^{-1}): $K_p = c_p/(c_g \cdot M)$, where M (μg m^{-3}) is the aerosol mass concentration. When the gas/particulate partitioning is dominated by absorption into the aerosol, the partitioning coefficient K_p for the compound is given by (Pankow, 1994).

$$K_p = \frac{N_s a_{tsp} T e^{(Q_l/Q_v)/RT}}{1600\, p_L^o},$$

T (K) is the temperature and p_L^o (torr/Pa) is the vapour pressure of the compound as a (sub-cooled/super-cooled) liquid. The relationship between p_L^o and T is expressed by the Clapeyron-Clausius equation as $p_L^o \sim \exp(-Q_v/RT)$ where Q_v is the enthalpy of vaporization of the sub-cooled (super-cooled) liquid and R is the gas constant. The temperature dependence of the partitioning coefficient K_p therefore follows the below temperature dependency (Takekawa et al., 2003):

$$K_p = K_{p,298} \cdot \frac{T}{298} \cdot \exp\left[\frac{Q_v}{R}\left(\frac{1}{T} - \frac{1}{298}\right)\right], \qquad \text{--- (1)}$$

where $K_{p,298}$ is the value of K_p at 298 K.

The corresponding values $K_{p,298}$ and Q_v of anthraquinone and anthracene are derived from the regression of these parameters in Equation 1 based on in situ observation of the particulate mass fraction. The temperature T and aerosol mass concentration M corresponding to each individual observation record are obtained from the EAC4 (ECMWF Atmospheric Composition Reanalysis 4) global reanalysis dataset (Inness et al., 2019), since those parameters are usually not known for every observation. For consistency, the EAC4 dataset is also used for calculating the gas/particulate fractions in the MOZART-4 model (see Equation 2). The sum of all fine-mode (0.03 – 0.5 μm) aerosols concentrations of the EAC4 datasets is used for the aerosol mass concentration M.

Based on the best fit of the measured particulate mass fraction, the values of $K_{p,298}$ = 6.76 x 10^{-4} m^3 μg^{-1} and Q_v = 30.8 kJ mol-1 are obtained for anthracene, and the values of $K_{p,298}$ = 1.48 x 10^{-2} m^3 μg^{-1} and Q_v = 43.3 kJ mol^{-1} are obtained for anthraquinone.

Combining the expression of θ and K_p, the particulate mass fraction θ is obtained from:

$$\theta = \frac{K_p M}{1 + K_p M}. \qquad \text{--- (2)}$$

As the upper decile (quartile) of mass fraction in particulate phase for anthracene over the global land surface is less than 2.5% (1%), all anthracene is assumed to be in gaseous phase in all of our simulations.

The mass fraction in particulate phase for anthraquinone plays a role in the parameterization of dry deposition and wet scavenging of anthraquinone, which is described in Section (5) and (6).

Production and Destruction of Anthraquinone in the Atmosphere

To calculate the global atmospheric distribution of anthraquinone, we account for both its direct surface emissions as well as for its production from the photo-oxidation of anthracene (ANT), initiated by the hydroxyl radical OH:

$$\text{ANT} + \text{OH} \rightarrow \ \dots \ \rightarrow \gamma\ \text{AQ},$$

with a rate constant of 1.29 × 10^{-10} cm^3 $molecules^{-1}$ s^{-1} (Goulay et al., 2005; Manion et al., 2008; McGillen et al., 2020). The EAC4 dataset for OH concentrations of the year 2014 is used as an input to calculate the above chemical transformation of anthracene from anthraquinone. A second-order chemical reaction between anthracene and OH is considered, with a rate constant of 1 × 10^{-10} cm^3 $molecules^{-1}$ s^{-1}. A factor of 0.95 is applied to the rate constant to account for the slowing down of the reaction for particulate-phase anthracene, which can reach 5% of the total anthracene content in highly-polluted regions.

The yield of anthraquinone formation from this reaction (γ) is unknown and working values adopted in our model are informed by the chemistry of similar chemical species. Quantum calculations on the atmospheric oxidation of gaseous anthracene initiated by OH radicals (Zeng et al., 2020) show that the production yield of the two intermediate products 2,10-hydroxyanthrone and 10-hydroperoxyanthrone is within a range of 50 – 85%, depending on the NO_X concentration level. As these two compounds are expected to be oxidized further to anthraquinone (while perhaps also being partially lost through deposition), we adopt for the formation yield of anthraquinone an upper value of 50%, referred as production scenario Y1 (see Table 1). This scenario is adopted in our simulations discussed in the Main Text (M1-3). On the other hand, Wang et al. (Wang et al., 2007), for example, indicate that the yield for the formation of 9,10-phenanthrenequinone from the atmospheric gas-phase oxidation of phenanthrene (an isomer of anthracene) is only around 3%. In presence of catalysts such as nitric acid, for example on the interface layer on particles (Chen and

Zhu, 2014), the production yield of anthraquinone from anthracene can be enhanced (Das and Das, 1982). We adopt therefore in our model a value of 10% referred to as production scenario Y2. The large uncertainty on the chemical formation of anthraquinone is expected to have limited effects on the calculated atmospheric concentrations, because the contribution of direct emissions usually dominates.

We consider the destruction of anthraquinone by several mechanisms, the rate of which should all be considered as uncertain. One of them is provided by the photo-oxidation by the OH radical (Scenario D1). The corresponding rate constant has been reported in the particle-phase to be 8.2 $\times 10^{-14}$ cm^3 molecule^{-1} s^{-1} (Miet et al., 2014). A gas-phase value of 1.5 x 10^{-12} cm^3 molecule^{-1} s^{-1} has also been estimated (United States Environmental Protection Agency, 2011). The corresponding destruction time constant is about 70 days for the particle-phase destruction and of the order of 3 days for the gas-phase reaction. In the absence of further information, and assuming a partitioning of gas phase to particle phase of 25/75, we combine these rate coefficients to estimate an overall destruction time scale for anthraquinone by the OH reaction of close to 10 days. Although this lifetime is uncertain, its precise value is usually not very important due to the existence of other sinks (see below).

The second destruction mechanism is photolysis. The Hazardous Substance Data Bank reports a lifetime of anthraquinone against atmospheric photolysis of 9 minutes in aqueous solution (U.S. National Library of Medicine, 2006). The lifetime against photolysis of naphthoquinone, an organic species that shares a chemical structure similar to that of anthraquinone, but with only two six-membered rings, is estimated to be around 2 hours in the atmosphere (Atkinson et al., 1989). We consider a scenario in which the photolysis time constant is 20 minutes at all altitudes when the Sun is directly overhead (Scenario D2). In this case, the dependency of the photolysis frequency with the solar zenith angle is assumed to be similar to that of the photolysis frequency of NO_2. Two more scenarios similar to Scenario D2 with a photolysis time constant of 40 minutes (Scenario D3) and 1 hour (Scenario D4) are also considered in other simulations.

Surface deposition of anthracene and anthraquinone

The surface uptake of anthracene and anthraquinone is expressed as a function of surface properties. Over the continent, we assume that anthracene is deposited on the surface with an effective velocity of 0.24 cm s^{-1}, based on the dry deposition velocity derived for 3-ring PAHs in both gas and particle phase (Chang et al., 2003). Over water surfaces, we imposed a lower deposition velocity of 0.05 cm s^{-1}, as the solubility of anthracene is low (Yalkowsky et al., 2016).

The deposition velocity for gaseous-phase anthraquinone $v_{dep,AQ,g}$ (cm s^{-1}) is a function of θ and the fraction of land use categories in a grid:

$$v_{dep,AQ,g} = f_{veg} \cdot 0.7 + f_{water} \cdot 0.5 + f_{soil} \cdot 0.05 \,,$$

where f_{veg}, f_{water} and f_{soil} are the fractions covered by vegetation, water and bare soil in a grid. These three land surface categories are constituted from combining some of the 11 MOZART land use types at a spatial resolution 1° x 1° (see Table S6a & S6b). The deposition velocity for particulate-phase anthraquinone $v_{dep,AQ,p}$ equals to 0.15 cm s^{-1} in every grid.

The information on AQ uptake being very limited, we derive a value for the deposition velocity based on measurements made for other gas-phase organic compounds with similar chemical properties. Using the model of Wesely (Wesely, 2007) with Henry's law constants and saturation vapor pressures derived using group estimation methods, Hodzic et al. (Hodzic et al., 2013) indicated that the dry deposition velocity of organic vapors with two functional groups and a saturation vapor pressure of ~1 µg/m^3 (similar to anthraquinone) should lie between 0.2 and 3.5 cm s^{-1}. Using a similar approach constrained by available measurements for oxygenated organic compounds, Müller et al. (Müller et al., 2018) derived daily averaged deposition velocities of ~0.7

cm s^{-1} for hydroxyacetone, which has a Henry's law constant similar to that of anthraquinone (of the order of 10 to 10^2 mol m^{-3} Pa^{-1}) (Lee and Zhou, 1993; U.S. National Library of Medicine, 2006). This value is very close to the respective deposition velocities of 0.74 cm s^{-1} measured by Nguyen et al. (Nguyen et al., 2015) using the eddy covariance technique at a forested site in Alabama. We therefore estimate the deposition velocity of gas-phase anthraquinone to be 0.7 cm s^{-1} over vegetated land.

For anthraquinone in the particle phase, the deposition velocity depends highly on the size of the particles on which anthraquinone condenses. The dry deposition velocities of submicron and coarse-mode particles are typically 0.1 and 1 cm s^{-1}, respectively (Reddy et al., 2005; Hodzic et al., 2013) Most of the anthraquinone is condensed on submicron particles because of the relatively large molecular weight of this molecule (Tasdemir and Esen, 2007). We therefore estimate the deposition velocity to be 0.15 cm s^{-1} for anthraquinone in the particle phase over vegetated land.

Wet scavenging of anthraquinone

As anthracene resides mostly in the gaseous phase and its solubility in water is low, the wet scavenging for anthracene is neglected in all of the simulations.

Depending on the temperature-dependent particulate mass fraction of anthraquinone (θ), the removal of anthraquinone by rainout with a loss rate W (s^{-1}) is expressed as (Brasseur et al., 1998):

$$\frac{dAQ}{dt} = -W \cdot AQ\ ,$$

with $W = \theta \cdot W_p + (1-\theta) \cdot W_g$, where W_g and W_p are the loss rates for gaseous and particulate anthraquinone respectively.
The rate of in-cloud scavenging for gaseous anthraquinone is given by (Giorgi and Chameides, 1985):

$$W_g = \frac{W_{\mathrm{H2O}}}{X_{\mathrm{H2O}} + A/HRT}\ ,$$

where W_{H2O} is the rainwater tendency (g cm^{-3} s^{-1}), X_{H2O} is the mass density of convective and non-convective raindrops (g cm^{-3}), A is the Avogadro number, R is the perfect gas constant (= 8.205×10^{-2} atm cm^{-3} K^{-1} M^{-1} g^{-1}), T is the temperature (K), and H is the effective Henry's law, which is equal to 4.3×10^4 M atm^{-1} for anthraquinone (U.S. National Library of Medicine, 2006).

For particulate anthraquinone, the loss rate W_p is set to be 20% of the scavenging rate of HNO_3.

Re-volatilization

Semi-volatile compounds deposited on the soil or on vegetation can undergo re-volatilization (Smit et al., 1997; Smit et al., 1998). Several parameterizations exist. The Henry's Law constant for anthraquinone is estimated as 420 mol m^{-3} Pa^{-1} (U.S. National Library of Medicine, 2006). This estimated value indicates that anthraquinone is expected to be essentially nonvolatile from water surface (Lyman et al., 1990). With its vapor pressure of 1.16×10^{-7} mm Hg (Shimizu et al., 1987), anthraquinone is not expected to volatilize from soil surfaces (U.S. National Library of Medicine, 2006).

From the parametrization of (Smit et al., 1998), the cumulative volatilization of a semi-volatile compound from plants is related to its vapor pressure (*VP* in unit of mPa). Considering all

investigated crops presented in (Smit et al., 1998), the cumulative volatilization (*CV*, in percentage of the deposited amount) after 7 days of deposition is given by:

$$\log_{10} CV = c + d \log_{10} VP \, ,$$

where c = 1.528 and d = 0.466. With VP = 0.0155 mPa at 25ºC for anthraquinone (Bardi et al., 1973), the corresponding cumulative volatilization is 4.8 %. Given that the estimated fraction is relatively low, and the above parametrization could even overestimate the fraction due to other processes such as phototransformation or hydrolysis (Smit et al., 1998), the re-volatilization of anthraquinone from plants, as well as from other surfaces, is therefore neglected in this study.
Given the higher gas-phase fraction and higher vapor pressure of anthracene, its cumulative volatilization could be significantly larger than that of anthraquinone. However, given that its chemical transformation rate is relatively fast, anthracene deposition plays only a small role as a sink and thus the subsequent re-volatilized amount of anthracene would be insignificant compared to direct emissions. Therefore, the re-volatilization of anthracene is also not considered in this study.

Model settings of sensitivity cases that are not discussed in the Main Text (Case S1-8)

Unlike the simulations discussed in the Main Text, the sensitivity cases S1-8 adopt simplistic treatment for the gas/particulate partitioning, i.e. the gas-phase fraction of anthraquinone is assumed constant (25/75, or θ = 0.75) and anthracene resides entirely in the gaseous phase.

For the secondary formation of anthraquinone from the photooxidation of anthracene, a first-order chemical reaction with a chemical lifetime of 1 hour is implemented for this reaction during daytime, corresponding to a OH concentration of 2×10^6 molecules cm^{-3}, the corresponding chemical lifetime of anthracene is about 1 hour. At night, with little OH present in the atmosphere, but a possible slow oxidation of anthracene by ozone or other oxidants, we adopt a reaction time constant of 10 hours.

For surface deposition of anthraquinone, assuming a partitioning of gas phase to particle phase of 25/75, we adopt an overall deposition velocity of 0.3 cm s^{-1} over vegetated land. The deposition velocities over other types of land surfaces are adjusted accordingly as shown in Table S6a. Wet scavenging of both gas and particulate anthraquinone is not considered.

The different scenarios and model settings of all the sensitivity cases in this study (M1-3 & S1-8) are summarized in Table S7.

Model comparison with observational data

Modelled concentrations of anthraquinone are compared with observational data gathered from existing literature (see Table S1 and S2). These observational sites are classified into non-background and background sites. There are in total 100 measurements conducted in non-background sites located in urban areas and near emission sources such as road traffic or power plants, and 49 measurements conducted in the background sites located in suburban or rural areas. Monthly averages of the modelled concentrations (M_i) over the month(s) in which the measurement

was conducted are used to compare with the corresponding observed data (O_i). The following two evaluation metrics are adopted:

$$\textbf{Normalised mean bias } NMB = \frac{\sum_{i=1}^{N}(M_i - O_i)}{\sum_{i=1}^{N} O_i}$$

$$\textbf{Pearson correlation coefficient } r = \frac{\sum_{i=1}^{N}(M_i - \bar{M})(O_i - \bar{O})}{[\sum_{i=1}^{N}(M_i - \bar{M})^2]^{\frac{1}{2}}[\sum_{i=1}^{N}(O_i - \bar{O})^2]^{\frac{1}{2}}}$$

The different scenarios and modelling settings adopted in all sensitivity cases are listed in Table S7. The results of the model validation for all sensitivity cases, including the normalized mean bias (*NMB*) and the Pearson correlation coefficient (*r*) are shown in Table S8.

As discussed in the Main Text, the model shows reasonable agreement with the observation data, but generally underestimates the concentration in all simulations. Most of the sensitivity cases also manage to capture the substantially higher anthraquinone concentrations observed in winter. However, the enhanced seasonal variation (E2 and E3, i.e., Cases S2, S3 and S5-8) clearly improves the model performance in both the bias and correlation against the observed data. The cases with the higher AQ production yield Y1 (i.e., Cases S6 and S8) show better agreement with the observation compared to the lower-yield cases. There is also a possibility that the anthraquinone emissions used in the simulations are underestimated.

Regression of the temperature-dependent gas/particulate partitioning of anthraquinone and anthracene

To derive the temperature-dependent gas/particulate partitioning of anthraquinone and anthracene, the parameters $K_{p,298}$ and Q_v in Equation 3 were derived using linear regression. The measured mass fraction in particulate phase θ, and the temperature *T* and aerosol mass concentration *M* of the individual measurements are used in fitting the best regression model (labelled as *fit obs* in Figure S5). Since the ambient temperature and aerosol loading were recorded in only 7 of the measurements of the particulate mass fraction, two additional regression fits were performed using (*T*,*M*) from the CAMS global reanalysis (EAC4) dataset (Inness et al., 2019). The first fit uses the CAMS PM10 concentration for *M* (labelled as *fit CAMS PM10* in Figure S5), the second set uses the total concentration of fine-mode aerosols (labelled as *fit CAMS aerosols* in Figure S4). The best-fit values of $K_{p,298}$ and Q_v from these regression models are listed in Table S9. For comparison, the values of $K_{p,298}$ from the models of Mackay (Mackay et al., 1986) and Octanol/air (Koa) (Finizio et al., 1997), as obtained from the EPI suite (United States Environmental Protection Agency, 2011), are also listed.

As seen in Table S9, the best-fit values of Q_v are all close to the value of 42 kJ mol^{-1} for Secondary Organic Aerosol derived in the laboratory (Offenberg et al., 2006). The best-fit values of $K_{p,298}$ from the 3 regression models are of the order of 0.01 m^3 µg^{-1} and they all lie within a factor of two of each other. In contrast, the $K_{p,298\ \mathrm{value}}$ from the Mackay and Koa models are in the order of 0.0001 m^3 µg^{-1}, i.e. 2 orders of magnitude smaller than the fitted $K_{p,298}$. The dependency of the resulting θ from the 5 models on *T* and *M* are plotted in Figure S6. The low values of θ from the two EPI suite models are inconsistent with the existing measurements. Therefore, we adopted the fitted $K_{p,298}$ in our simulations instead of those from the conventional multimedia models. The fit based on CAMS fine-mode aerosol data can be considered as the best regression model among the three, as its results are close to those from the fit based on observed *T* and *M* (*obs fit*) and it relies on a larger dataset. Therefore, the best-fit values $K_{p,298} = 1.48 \times 10^{-2}$ m^3 µg^{-1} and $Q_v = 43.3$ kJ mol^{-1} are used in our simulation. The resulting global distribution of the monthly-averaged mass fraction in particulate phase θ of anthraquinone for the month of January and July are shown in Figure S7.

A similar approach was followed to estimate the parameters $K_{p,298}$ and Q_v for anthracene (see Figure S8). Apart from the aforementioned three fits, an additional fit based on the results from a measurement campaign in Chicago (Simcik et al., 1998) is performed, denoted as *fit Simcik* in Figure S8. The best-fitted values from these 4 regression models and the values from the Mackay and Koa models are listed in Table S10.

As seen on Table S10, the best-fit values of Q_v from the 4 regression models are between 20 - 30 kJ mol^{-1}. The best-fit values of $K_{p,298}$ from the first 3 regression models are of the order of 10^{-5} and 10^{-4}. This is around one magnitude larger than the values from the Mackay and Koa models. Among all models, the best-fit value of $K_{p,298}$ from *fit Simcik* is exceptionally high, of the order of 10^{-3}. The dependency of the resulting θ from the 5 models on T and M is plotted in Figure S9. Except for *fit Simcik*, all models predict θ lower than 10% in the range of T and M considered here. Because the *Simcik* data were obtained from a unique measurement campaign (Chicago), it may not be representative for the global distribution of θ. As for anthraquinone, we adopt the fit based on CAMS fine-mode aerosol (*fit CAMS aerosols*). The resulting global distribution of the monthly-averaged mass fraction in particulate phase θ of anthracene for the month of January and July are shown in Figure S10. Due to the low θ of anthracene in the typical atmospheric range of T and M around the globe, anthracene is assumed to be entirely in gaseous phase in all simulations.

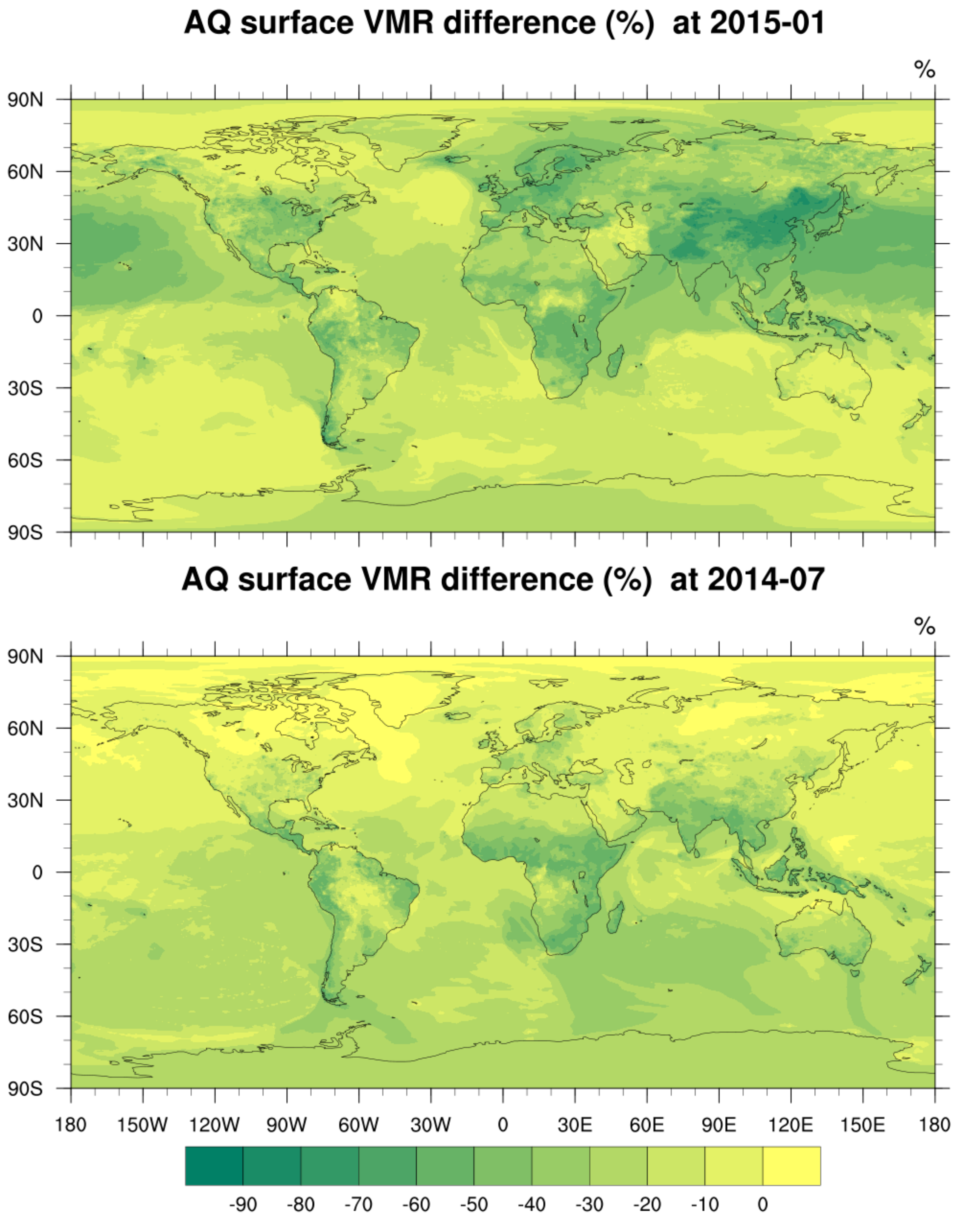

Figure S1. Reduction in anthraquinone surface concentrations (in percentage) in the month of January (upper panel) and July (below panel) due to a reduction of residential anthraquinone emissions (Case M3 minus Case M1).

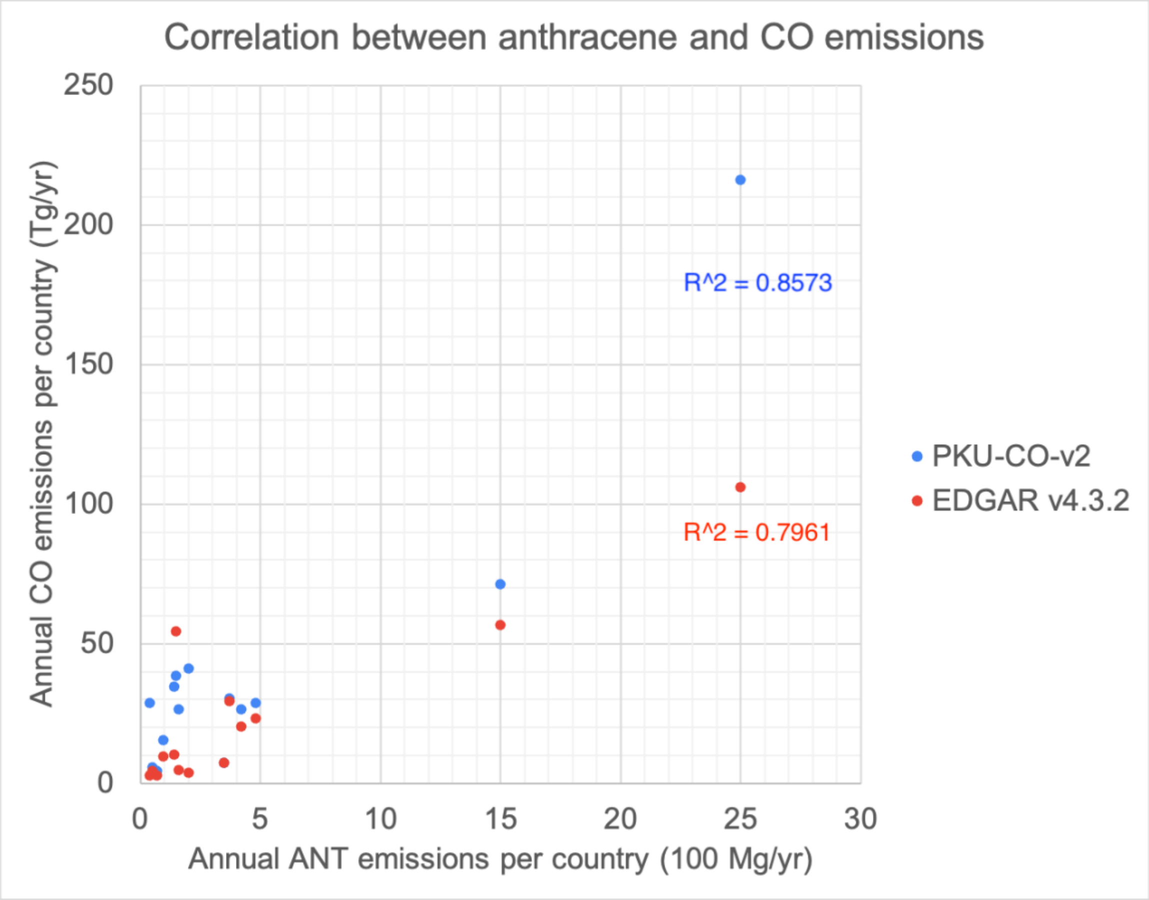

Figure S2. Correlation between anthracene and CO emissions. The annual anthracene (ANT) emissions per country is plotted against the annual CO emissions per country. The annual emissions of anthracene are from the reported values of the PKU-PAH inventory (Shen et al., 2013). For CO emissions, two emission inventories, namely PKU-CO-v2 (Zhong et al., 2017) and EDGAR v4.3.2 (Janssens-Maenhout et al., 2017), are considered here. The annual emissions of top emitting countries, including Brazil, China, Congo DR, Ethiopia, India, Indonesia, Myanmar (Burma), Nigeria, Russia, US, Germany, Japan, Mexico and Australia, are included in this plot. With both inventories, the emission of anthracene has a relatively high correlation with the total CO emissions.

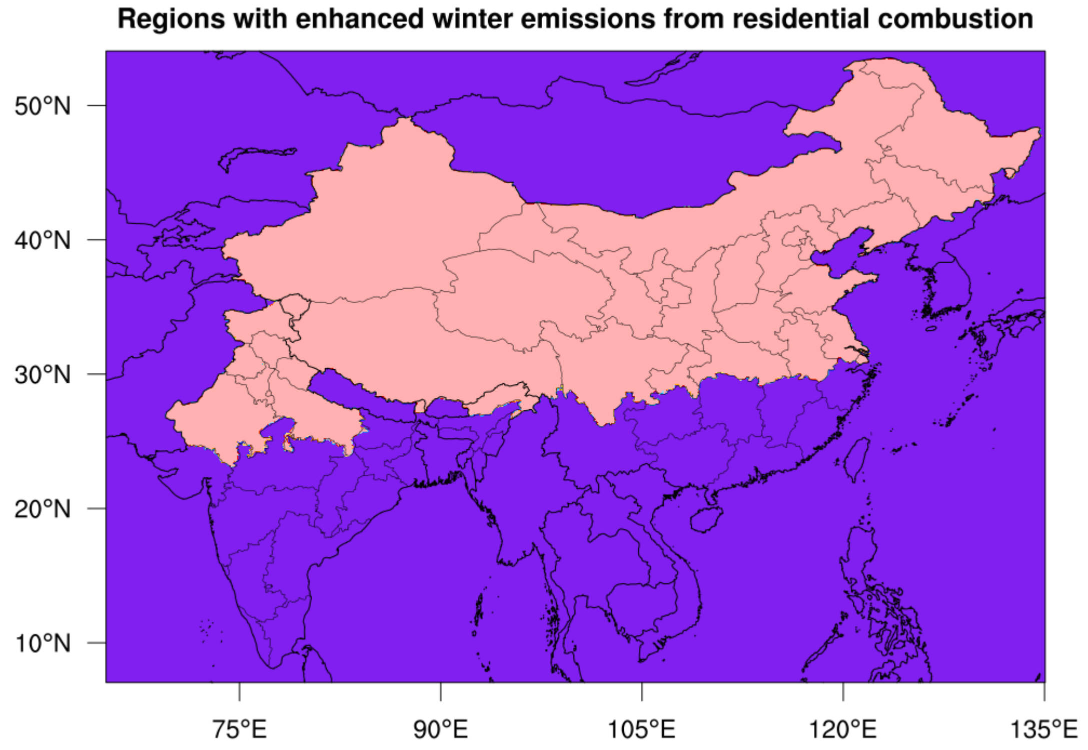

Figure S3. Map showing the regions with enhanced winter emissions from the sector of residential combustion in the emission scenario E3. These regions consist of the Chinese provinces located north of the Yangtze River (including Xinjiang, Gansu, Inner Mongolia, Heilongjiang, Jilin, Liaoning, Beijing, Tianjin, Hebei, Shandong, Shanxi, Shaanxi, Ningxia, Qinghai, Tibet, Sichuan, Chongqing, Hubei, Anhui, Jiangsu and Shanghai) and the Northern India provinces including Jammu and Kashmir, Ladakh, Himachal Pradesh, Punjab, Uttarakhand, Haryana, Rajasthan, Delhi, Uttar Pradesh, Sikkim and Arunachal Pradesh.

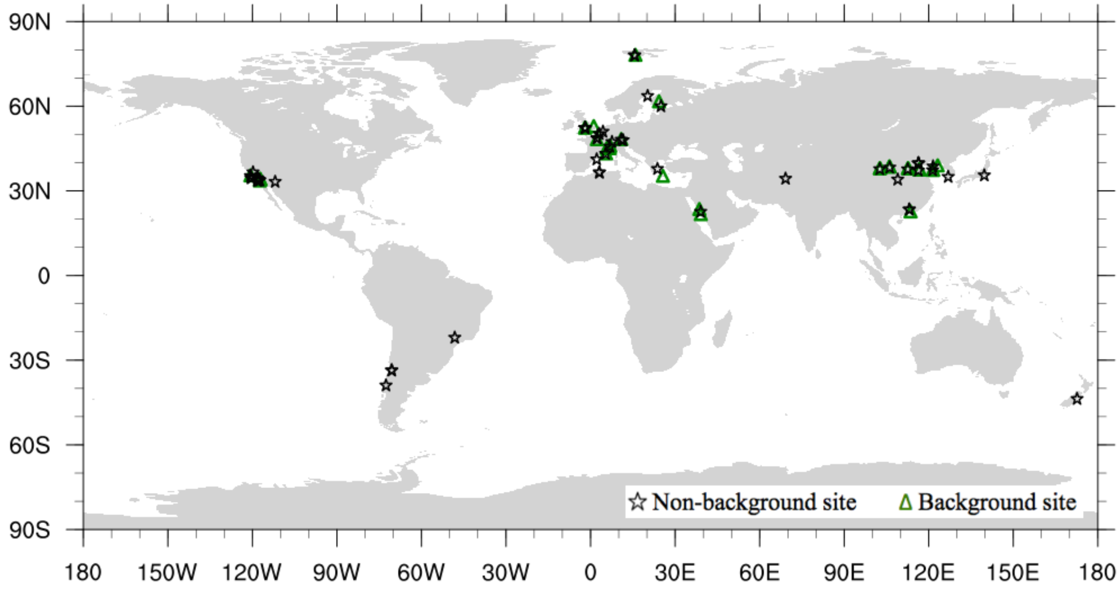

Figure S4. Map showing the locations of the in-situ measurements based on existing literature listed on Table S1 and S2.

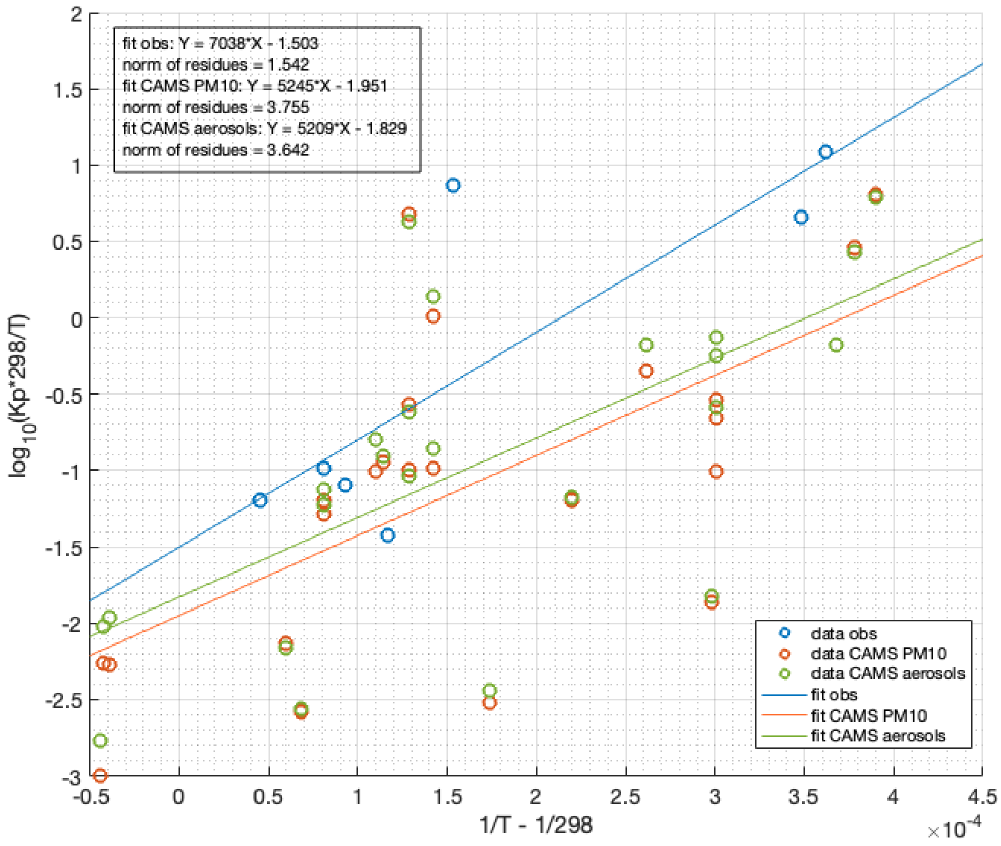

Figure S5: Parameter search for $K_{p,298}$ and Q_v in Equation 3 for anthraquinone using measured mass fraction in particulate phase (*θ)* based on measurements, ambient temperature *T* from the corresponding measurements or from the EAC4 datasets, and the aerosol mass concentrations *M* from the measurements or the EAC4 datasets. Note that K_p is a function of *θ* and *M*. Here, the results of the regression models (*fit obs*, *fit CAMS PM 10* and *fit CAMS aerosols*) are shown. Please refer to the Supplementary Text for the definition of the regression fits. The best-fit values of $K_{p,298}$ and Q_v are shown in Table S9.

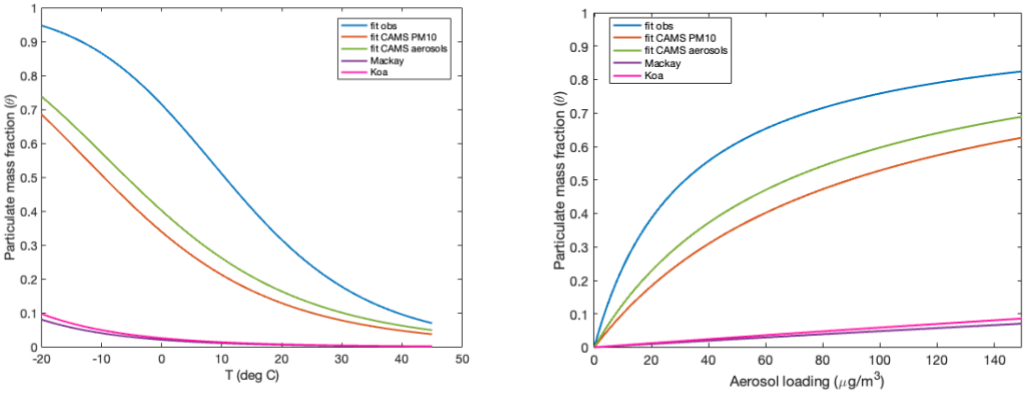

Figure S6: The dependency of the particulate mass fraction θ of anthraquinone on ambient temperature T (left panel) and aerosol mass concentration M (right panel) of the models shown in Table S9. On the left panel, the value of M is set at 10 μg/m^3. On the right panel, the value of T is set at 25°C.

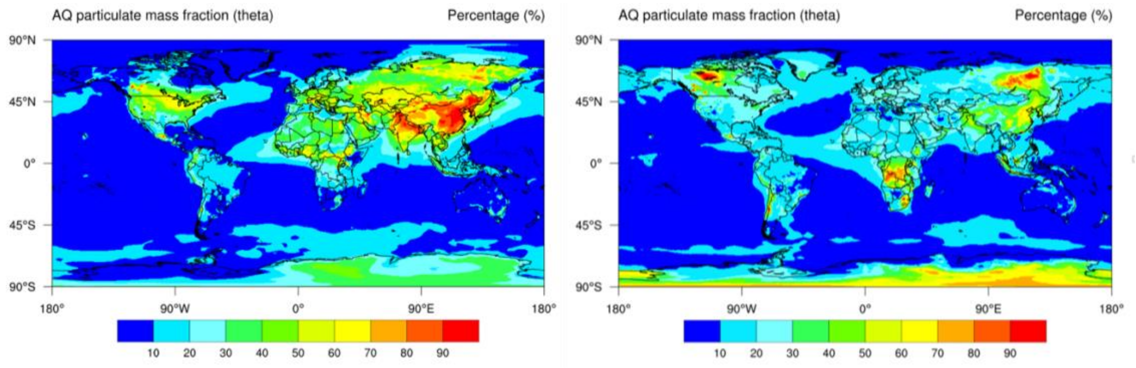

Figure S7: Global distribution of the monthly-averaged mass fraction in particulate phase θ of anthraquinone for the month of January (left panel) and July (right), using the best-fit values $K_{p,298}$ = 1.48 x 10^{-2} m^3 μg^{-1} and Q_v = 43.3 kJ mol^{-1} adopted in the model simulations.

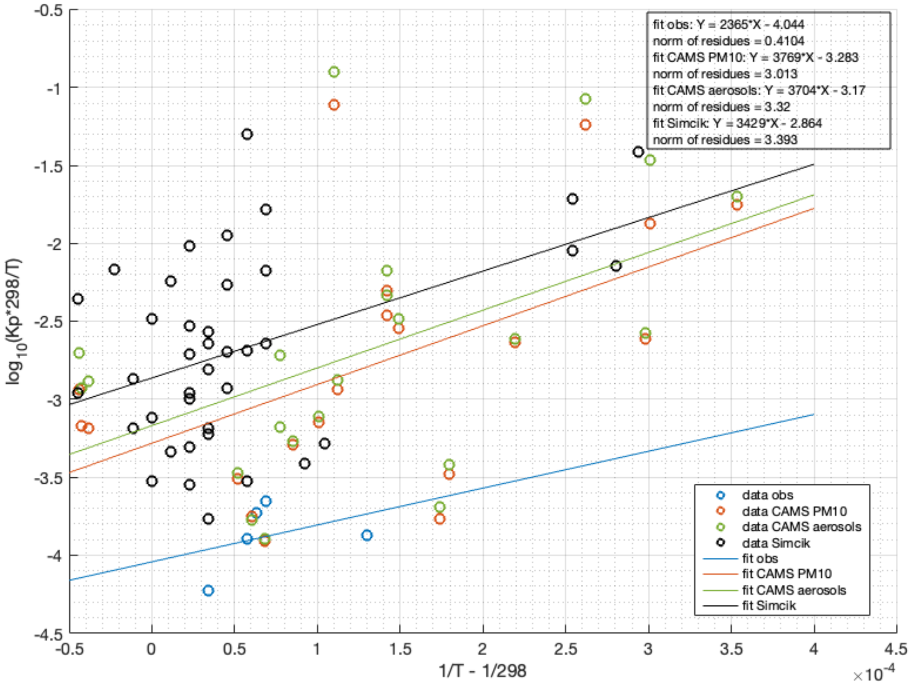

Figure S8: Parameter search of for $K_{p,298}$ and Q_v in Equation 3 for anthracene using measured particulate mass fractions θ, ambient temperature T from either the corresponding measurements or the EAC4 datasets, and the aerosol mass concentrations M from the corresponding measurements or the EAC4 datasets. Note that K_p is a function of θ and M. Here, the results of the regression models (*fit obs*, *fit CAMS PM 10*, *fit CAMS aerosols* and *fit Simcik*) are shown. Please refer to the Supplementary Text for the definition of the regression fits. The best-fit values of $K_{p,298}$ and Q_v are shown in Table S10.

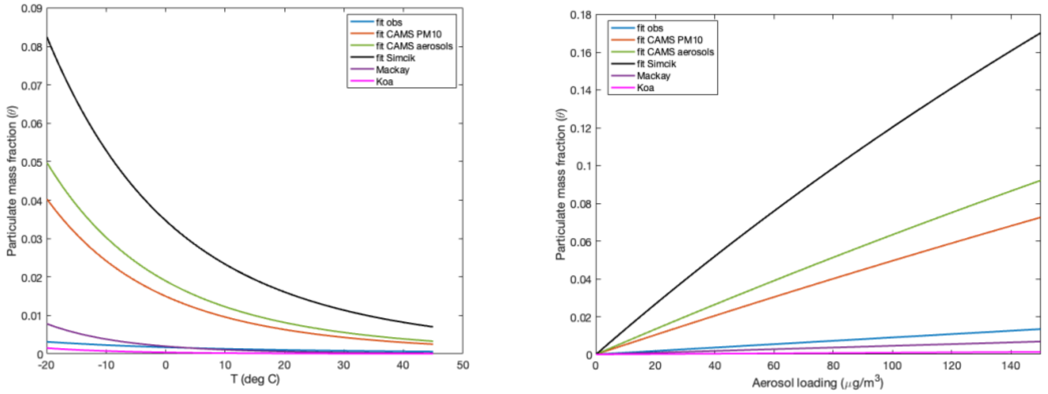

Figure S9: The dependency of the particulate mass fraction θ of anthracene on ambient temperature T (left panel) and aerosol mass concentration M (right panel) for the models shown in Table S10. On the left panel, the value of M is set at. 10 µg/m^3. On the right panel, the value of T is set at 25°C.

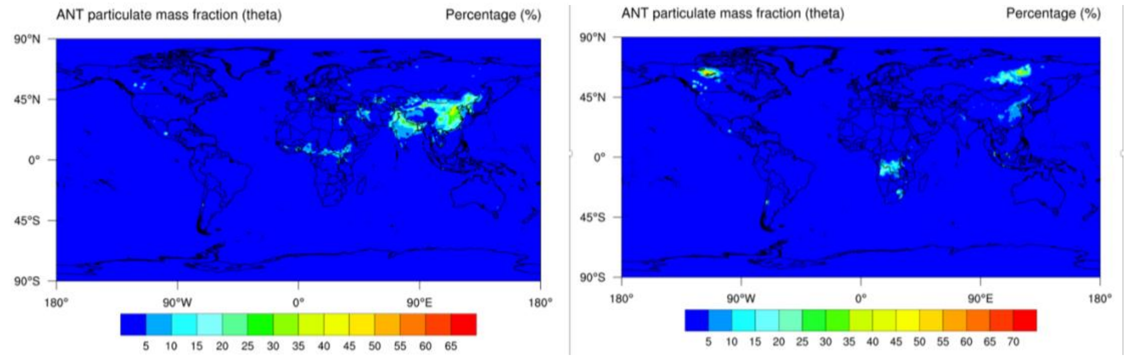

Figure S10: Global distribution of the monthly-averaged mass fraction in particulate phase θ of anthracene for the month of January (left) and July (right), using the best-fit values $K_{p,298}$ = 6.76 x 10^{-4} m^3 μg^{-1} and Q_v = 30.8 kJ mol^{-1}.

Table S1. List of non-background sites of surface anthraquinone concentration measurement. The measured concentrations are given in the unit of ng/m^3. AQ (g), AQ (p) and AQ (t) refer to the measured anthraquinone concentration in gaseous, particulate and both phases respectively.

Location	Landuse	Time period	AQ (g)	AQ (p)	AQ (t)	Reference
**East Asia**						
PKU, Beijing, China	Urban	28 Jul-20 Sep 2008 Source-control period (Olympics 2008)		0.21		(Wang et al., 2011)
PKU, Beijing, China	Urban	21 Sep – 7 Oct 2008 Non source-control period (after Olympics 2008)		0.323		(Wang et al., 2011)
Beijing, China	Urban	Apr - Oct 2012		1.17		(Lin et al., 2015)
IAP, Beijing, China	Urban	9 Nov - 11 Dec 2016		5.12		(Lyu et al., 2019)
Qingyuan, Guangdong, China	Industrial	Aug 2009		0.07		(Wei et al., 2012)
Qingyuan, Guangdong, China	Industrial	Aug 2009		0.10		(Wei et al., 2012)
Qingyuan, Guangdong, China	Industrial	Aug 2009		0.11		(Wei et al., 2012)
Qingyuan, Guangdong, China	Industrial	Aug 2009		0.15		(Wei et al., 2012)
Qingyuan, Guangdong, China	Industrial	Jan - Feb 2010		0.23		(Wei et al., 2012)
Qingyuan, Guangdong, China	Industrial	Jan - Feb 2010		0.29		(Wei et al., 2012)
Qingyuan, Guangdong, China	Industrial	Jan - Feb 2010		0.24		(Wei et al., 2012)
Qingyuan, Guangdong, China	Industrial	Jan - Feb 2010		0.20		(Wei et al., 2012)
Wuwei. China	Urban	Apr 2010 - Mar 2011			13.00	(Li et al., 2015)
Yinchuan, China	Urban	Apr 2010 - Mar 2011			7.80	(Li et al., 2015)
Taiyuan, China	Urban	Apr 2010 - Mar 2011			17.00	(Li et al., 2015)
Beijing, China	Urban	Apr 2010 - Mar 2011			7.70	(Li et al., 2015)
Dezhou, China	Urban	Apr 2010 - Mar 2011			13.00	(Li et al., 2015)

Yantai, China	Urban	Apr 2010 - Mar 2011			13.00	(Li et al., 2015)
Dalian, China	Urban	Apr 2010 - Mar 2011			5.20	(Li et al., 2015)
Xian, China	Mixed	Mar 2012	4.08	3.90	7.98	(Wei et al., 2015)
Xian, China	Mixed	Sep 2012	3.83	4.35	8.18	(Wei et al., 2015)
Xian, China	Mixed	Heating period in 2013		13.70		(Wang et al., 2016)
Downtown Tokyo, Japan	Urban	31 Jul -7 Aug 2007	0.08			(Kojima et al., 2010)
Downtown Tokyo, Japan	Urban	22 -31 Jan 2008		0.52		(Kojima et al., 2010)
Gwangju, South Korea	Urban	26 Mar - 4 May 2001		0.73		(Park et al., 2006)
**Middle East and Africa**						
Kabul, Afghanistan	Urban	19 Oct - 2 Nov 2009		2.40		(Wingfors et al., 2011)
Rabegh, Saudi Arabia	Residential	Feb-Apr 2013			4.02	(Harrison et al., 2016)
Rabegh, Saudi Arabia	Residential	30 Sep 2013			6.50	(Harrison et al., 2016)
Algiers, Algeria	Urban	Aug 1998			6.20	(Yassaa et al., 2001)
Algiers, Algeria	Urban	Feb 1999		1.00		(Yassaa et al., 2001)
Oued Smar landfill, Algeria	Landfill	Aug 1998			1.50	(Yassaa et al., 2001)
Oued Smar landfill, Algeria	Landfill	Feb 1999		6.20		(Yassaa et al., 2001)
**Europe**						
Antwerp, Belgium	Residential	Jan 1976		0.98		(Cautreels et al., 1977)
Helsinki. Finland	Urban	9 Jul 2002		0.21		(Shimmo et al., 2004)
Helsinki, Finland	Urban	1 Feb 2003		0.64		(Kallio et al., 2003)
Douai, France	Urban	Winter		0.08-0.66		(Mirivel et al., 2010)
Les Bossons, French Alps, France	Traffic	15-22 Jan 2003		3.60		(Albinet et al., 2008)

Les Bossons, French Alps, France	Traffic	4-11 Jul 2004			0.97	(Albinet et al., 2008)
5 avenues, Marseille, France	Urban	22-29 Jul 2004			1.40	(Albinet et al., 2007)
Paris, France	Urban	72h total sampling time			70.00	(Nicol et al., 2001)
Paris France	Traffic	non-specific			0.66	(Ringuet et al., 2012)
Parc des Princes Tunnel (Paris, France)	Traffic	Jun 2013		0.20		(Keyte et al., 2016)
Augsburg, Germany	Urban	20 Aug - 17 Sep 2002		0.47		(Schnelle-Kreis et al., 2005)
Augsburg, Germany	Urban	24 Sep - 30 Nov 2002		1.46		(Schnelle-Kreis et al., 2005)
Augsburg, Germany	Urban	Mar- May 2003-2004		0.78		(Schnelle-Kreis et al., 2007)
Augsburg, Germany	Urban	Jun -Aug 2003-2004		0.20		(Schnelle-Kreis et al., 2007)
Augsburg, Germany	Urban	Sep - Nov 2003-2004		0.66		(Schnelle-Kreis et al., 2007)
Augsburg, Germany	Urban	Dec - Feb 2003-2004		1.76		(Schnelle-Kreis et al., 2007)
Augsburg, Germany	Urban	28 Jul - 3 Aug 2004	0.05	1.30	1.35	(Liu et al., 2006)
Munich, Germany	Urban	Non-specific		0.51-1.47		(Schnelle-Kreis et al., 2001)
Munich, Germany	Urban	Winter		1.46		(Schnelle-Kreis et al., 2005)
Munich, Germany	Suburb	All seasons 1996-1998		0.96		(Schnelle-Kreis et al., 2001)
Athens, Greece	Urban	Non-specific		0.97		(Valavanidis et al., 2006)
Longyearbyen powerplant, Svalbard, Norway	Power plant	27 Sep – 2 Oct 2018			15.76	(Drotikova et al., 2020)
UNIS, Svalbard, Norway	Urban	28 Sep-2 Oct 2018			5	(Drotikova et al., 2020)

Barcelona, Spain	Urban	Winter		0.07-0.31		(Castells et al., 2003)
Barcelona, Spain	Urban	Mar 1989			0.01	(Bayona et al., 1994)
Barcelona, Spain	Urban	Nov 1989			0.03	(Bayona et al., 1994)
Barcelona, Spain	Urban	Feb 1990			0.02	(Bayona et al., 1994)
Ume, Sweden	Traffic	14- 18 Apr 2009		0.05		(Wingfors et al., 2011)
Basel, Switzerland	Traffic	Autumn		0.03-2.68		(Niederer, 1998)
UK	Urban	72 h sample time		0.21		(Kelly et al., 1993)
Birmingham, UK	Traffic	For five days	0.98	0.09	1.07	(Delgado-Saborit et al., 2013)
Birmingham, UK	Traffic	Jan 2010			1.00	(Alam et al., 2013)
Queensway Road Tunnel (Birmingham, UK)	Traffic	Sep 2012			3.70	(Keyte et al., 2016)
Elms Road Observatory Site (Birmingham, UK)	Urban	Sep 2012			0.70	(Keyte et al., 2016)
BROS, Birmingham, UK	Traffic	3-20 Jan 2014	0.94	0.54		(Alam et al., 2015)
**North America**						
Tempe, AZ, US	Urban	Mar - Jun 2005			1.63	(Delhomme et al., 2008)
Atascadero, CA, US	Urban	over a 5-year period		0.18		(Eiguren-Fernandez et al., 2008)
Atascadero, CA, US	Urban	Spring, Summer, Winter 1995		0.11		(Manchester-Neesvig et al., 2003)
Fresno, CA, USA	Residential	Winter		0.47		(Chung et al., 2006)
Lompoc, CA, US	Urban	Spring, Summer, Winter 1995		0.03		(Manchester-Neesvig et al., 2003)
Lompoc, CA,US	Urban	over a 5-year period		0.12		(Eiguren-Fernandez et al., 2008)
Long Beach, CA, US	Urban	over a 5-year period	0.32	0.22	0.55	(Eiguren-Fernandez et al., 2008)

Long Beach, CA, US	Urban	Spring, Summer, Winter 1995		0.22		(Manchester-Neesvig et al., 2003)
Central Los Angeles, Azusa, and Claremont, CA, US	Urban	8-9 Sep 1993			0.30	(Fraser et al., 2000)
Mira Loma, CA, US	Urban	over a 5-year period		0.19		(Eiguren-Fernandez et al., 2008)
Mira Loma, CA, US	Urban	Spring, Summer, Winter 1995		0.12		(Manchester-Neesvig et al., 2003)
Riverside, CA, US	Urban	over a 5-year period		0.20		(Eiguren-Fernandez et al., 2008)
Riverside, CA, US	Urban	Spring, Summer, Winter 1995		0.16		(Manchester-Neesvig et al., 2003)
San Dimas, CA, US	Urban	over a 5-year period		0.25		(Eiguren-Fernandez et al., 2008)
San Dimas, CA, US	Urban	Spring, Summer, Winter 1995		0.14		(Manchester-Neesvig et al., 2003)
San Dimas, CA, US	Urban	May-Jul 2001		0.13		(Cho et al., 2004)
Upland, CA, US	Urban	over a 5-year period		0.24		(Eiguren-Fernandez et al., 2008)
Upland, CA, US	Urban	Spring, Summer, Winter 1995		0.20		(Manchester-Neesvig et al., 2003)
South America and Oceania						
Araraquara, Brazil	Industry	May-Jun 2010 (Day)		0.95		(Souza et al., 2014)
Araraquara, Brazil	Industry	May-Jun 2010 (Night)		0.73		(Souza et al., 2014)
Santiago, Chile	Urban	Aug 1998		0.12		(Tsapakis et al., 2002)
Santiago, Chile	Urban	Oct 1998		0.33		(Tsapakis et al., 2002)
Las Condes, Santiago, Chile	Urban	7-19 July 2000	0.67			(Maria del Rosario Sienra, 2006)
Las Condes, Santiago, Chile	Urban	28 Sep-7 Oct 2000		0.38		(Maria del Rosario Sienra, 2006)

Providencia, Santiago, Chile	Urban	7-19 July 2000	1.58			(Maria del Rosario Sienra, 2006)
Providencia, Santiago, Chile	Urban	27 Sep-7 Oct 2000		0.56		(Maria del Rosario Sienra, 2006)
Temuco, Chile	Urban	Sep 1998		0.24		(Tsapakis et al., 2002)
Christchurch, New Zealand	Urban	Winter		1.92		(Cavanagh, 2009)
Christchurch, New Zealand	Urban	Mar 2008 – Feb 2009			1.16	(Cavanagh, 2009)

Table S2. List of background sites of surface anthraquinone concentration measurements. The measured concentrations are given in the unit of ng/m^3. AQ (g), AQ (p) and AQ (t) refer to the measured anthraquinone concentration in gaseous, particulate and both phases respectively.

Location	Landuse	Time period	AQ (g)	AQ (p)	AQ (t)	Reference
East Asia						
Wuwei, China	Rural village	Apr 2010 - Mar 2011			3.30	(Li et al., 2015)
Yinchuan, China	Rural village	Apr 2010 - Mar 2011			15.00	(Li et al., 2015)
Taiyuan, Chian	Rural village	Apr 2010 - Mar 2011			12.00	(Li et al., 2015)
Dezhou, China	Rural village	Apr 2010 - Mar 2011			13.00	(Li et al., 2015)
Yantai, China	Rural village	Apr 2010 - Mar 2011			8.50	(Li et al., 2015)
Wuwei, China	Rural field	Apr 2010 - Mar 2011			12.00	(Li et al., 2015)
Yinchuan, China	Rural field	Apr 2010 - Mar 2011			7.50	(Li et al., 2015)
Taiyuan, China	Rural field	Apr 2010 - Mar 2011			13.00	(Li et al., 2015)
Dezhou, China	Rural field	Apr 2010 - Mar 2011			6.60	(Li et al., 2015)
Yantai, China	Rural field	Apr 2010 - Mar 2011			4.00	(Li et al., 2015)
Dalian, China	Rural field	Apr 2010 - Mar 2011			2.30	(Li et al., 2015)
Wanqingsham, Pearl River Delta, China	Rural	4 Nov - 6 Dec 2010	1.08	1.43	2.51	(Huang et al., 2014)
Middle East						
Abhur, Saudi Arabia	Suburb	Feb-Apr 2013			3.31	(Harrison et al., 2016)

Abhur, Saudi Arabia	Suburb	29 Sep 2013			8.30	(Harrison et al., 2016)
Rayes, Saudi Arabia	suburb	Feb-Apr 2013			3.15	(Harrison et al., 2016)
Rayes, Saudi Arabia	Suburb	1 Oct 2013			6.90	(Harrison et al., 2016)
**Europe**						
Hyytiälä, Finland	Rural	16 Mar - 10 Apr 2003		1.95		(Rissanen et al., 2006)
Hyytiälä, Finalnd	Rural	17 Mar - 10 Apr 2003		0.04		(Shimmo et al., 2004)
Paris, France	Suburb	not specified			0.056	(Ringuet et al., 2012)
Penne, Marseille, France	suburb	22 Jul 2004 – 29 Jul 2005			0.77	(Albinet et al., 2007)
Grenoble, France	Urban background	Feb 2013			1.47	(Tomaz et al., 2017)
Grenoble, France	Urban background	Dec 2013			2.40	(Tomaz et al., 2017)
Argentiere, French Alps, France	Rural	15-22 Jan 2003		0.57		(Albinet et al., 2008)
Argentiere, French Alps, France	Rural	4-11 Jul 2006			0.26	(Albinet et al., 2008)
Clos de l'Ours, French Alps, France	Suburb	15-22 Jan 2003		1.42		(Albinet et al., 2008)
Clos de l'Ours, French Alps, France	Suburb	4-11 Jul 2003			1.59	(Albinet et al., 2008)
Modane, France	Suburb	24-31 Jan 2005		2.76		(Albinet et al., 2008)
Modane, France	Suburb	25 Jun-2 Jul 2003			0.34	(Albinet et al., 2008)
Orelle, France	Rural	25 Jun-2 Jul 2003			0.37	(Albinet et al., 2008)

Sollieres, France	Rural	Winter 2002-2003 (7 sampling days of 12 h)	0.01	2.18		(Albinet et al., 2006)
Sollieres, France	Suburb	24-31 Jan 2006		2.36		(Albinet et al., 2008)
Sollieres, France	Suburb	25 Jun-2 Jul 2003			0.13	(Albinet et al., 2008)
Tigny, France	Rural	24-31 Jan 2003		1.77		(Albinet et al., 2008)
Tigny, France	Rural	25 Jun-2 Jul 2003			0.47	(Albinet et al., 2008)
Augsburg, Germany	Urban backgrounud	Summer 2005		0.39		(Sklorz et al., 2007)
Finokalia, Island of Crete, Greece	Marine background	17 – 21 Aug 2001		0.03		(Tsapakis et al., 2002)}
Adventdalen, Svalbard, Norway	Rural	28 Aug – 27 Sep 2018			0.07	(Drotikova et al., 2020)
Birmingham, UK	Urban backgrounud	Feb 2010			0.50	(Alam et al., 2013)
Elms Road Observatory Site (Birmingham, UK)	Suburb	3-20 Jan 2015	1.58	0.33		(Alam et al., 2015)
Weybourne Atmospheric Observatory, UK	Rural	2 Feb - 2 Mar 2010		0.06	0.06	(Alam et al., 2014)
Weybourne Atmospheric Observatory, UK	Rural	6 Aug – 2 Sep 2010		0.04	0.08	(Alam et al., 2014)
**North America**						
Alpine, CA, US	Rural	over a 5-year period	0.09	0.16	0.25	(Eiguren-Fernandez et al., 2008)
Alpine, CA, US	Rural	Spring, Summer, Winter 1995		0.08		(Manchester-Neesvig et al., 2003)
Atascadero, CA, US	rural	Jun 2001		0.14		(Cho et al., 2004)

Lake Elsinore, CA, US	Rural	over a 5-year period	0.09	0.19	0.28	(Eiguren-Fernandez et al., 2008)
Lake Elsinore, CA, US	Rural	Spring, Summer, Winter 1995		0.08		(Manchester-Neesvig et al., 2003)
Lake Arrowhead, CA, US	Rural	over a 5-year period	0.08	0.23	0.31	(Eiguren-Fernandez et al., 2008)
Lake Arrowhead, CA, US	Rural	Spring, Summer, Winter 1995		0.01		(Manchester-Neesvig et al., 2003)
Riverside, CA, US	suburb	May-Jul 2002		0.12		(Cho et al., 2004)

Table S3: Emission factors of anthracene (EF_{ANT}) and of anthraquinone (EF_{AQ}) for the residential sector, per fuel type (coal, crop and wood). The last column shows the ratio between the mean emission factors of anthraquinone and anthracene ($\frac{\overline{EF_{AQ}}}{\overline{EF_{ANT}}}$) for each combustion category. Values adopted from Shen (Shen, 2014).

Residential coal combustion

Fuel type	EF_{ANT} (mg/kg)	EF_{AQ} (mg/kg)	$\frac{\overline{EF_{AQ}}}{\overline{EF_{ANT}}}$
Honeycomb briquette, Beijing	0.1860	0.0066	0.2598
Honeycomb briquette, Taiyuan	0.2760	0.0320	
Raw chunk, Taiyuan	1.9450	1.3000	
Raw chunk-A, Yulin	14.9000	4.0000	
Raw chunk-B, Yulin	4.0500	0.2100	
Mean EF	4.2714	1.1097	

Residential crop combustion

Fuel type	EF_{ANT} (mg/kg)	EF_{AQ} (mg/kg)	$\frac{\overline{EF_{AQ}}}{\overline{EF_{ANT}}}$
Horsebean (Vicia faba)	0.9630	0.6500	0.6749
Peanut (Arachis hypogaea)	0.8800	1.1000	
Soybean (Cassia agnes)	0.6000	5.7000	
Cotton (Anemone vitifolia)	0.3750	0.6400	
Rice (Oryza sativa)	9.8700	1.2000	
Wheat (Triticum aestivum)	3.0900	1.8000	
Rape (Brassica napus)	2.9500	1.2000	
Sesame (Sesamum indicum)	1.0640	1.1000	
Corn (Zea mays)	1.0400	0.6700	
Mean EF	2.3147	1.5622	

Residential wood combustion

Fuel type	EF_{ANT} (mg/kg)	EF_{AQ} (mg/kg)	$\frac{\overline{EF_{AQ}}}{\overline{EF_{ANT}}}$
White Poplar	0.2300	0.0670	

Elm	0.3900	0.2100	0.8159
Locust	0.1300	0.1300	
Maple	0.2000	0.1700	
Fir	0.2700	0.1500	
Larch	0.1700	0.0690	
Water Chinese fir	0.2100	0.1500	
Cypress	-0.1600	0.1400	
Oak	0.1600	0.1500	
Chinese Pine	0.1400	0.0870	
Willow	0.0810	0.0830	
Paulownia tomentosa	0.1100	0.1500	
Toon	0.0840	0.0620	
White Birch	0.2700	0.1700	
Ribbed Birch	0.2200	0.1900	
Paulownia elongate	1.0000	0.6800	
Black Poplar	0.8800	1.3000	
China Aspen	0.3400	0.1900	
Chinaberry	0.4900	0.3600	
Jujube tree	0.3900	0.2000	
Persimmon tree	0.3400	0.2200	
Mulberry tree	0.3400	0.2500	
Peach tree	0.2600	0.1600	
Lespedeza	2.3000	1.7000	
Buxus sinica	1.2000	1.2000	
Holly	0.4900	0.3800	
Bamboo	0.5300	0.4100	
Mean EF	0.4098	0.3344	

Table S4: Mapping of the PKU-PAH anthracene emission sectors (Shen et al., 2013) to the emissions of the tagged model species.
ANT-ene, ANT-tra, ANT-res, ANT-bio refer to the tagged anthracene species emitted from the sectors of power generation and industry, traffic, residential combustion, and biomass burning, respectively, while AQ-ene, AQ-tra, AQ-res, AQ-bio refer to the tagged anthraquinone species from the same sources. See Main Text for details.

**Modelled species**	**Equivalent sector in PKU-PAH inventory**		**Mapping from PKU-PAH anthracene emission data (PKU-PAH-ANT)**
ANT-ene	Energy and industry sector combined		PKU-PAH-ANT (energy + industry)
ANT-tra	Transportation sector		PKU-PAH-ANT (transportation)
ANT-res	Residential sector		PKU-PAH-ANT (agricultural + deforestation)
ANT-bio	Agricultural and deforestation sectors combined		PKU-PAH-ANT (residential)
**Modelled species**	**Equivalent sector in PKU-PAH inventory**	**Assumption on emission relations**	**Mapping from PKU-PAH anthracene emission data (PKU-PAH-ANT)**
AQ-ene	Energy and industry	Coal burning	0.3 × [PKU-PAH-ANT (energy + industry)]
AQ-tra	Transportation	50% gasoline + 50% diesel vehicles	½ (0.50+0.65)×PKU-PAH-ANT (transportation)
AQ-res	Residential	50% crop burning + 50% wood burning	½ (0.67+0.82)×PKU-PAH-ANT (transportation)
AQ-bio	Agricultural and deforestation sectors	50% crop burning + 50% wood burning	½ (0.67+0.82)× [PKU-PAH-ANT (agricultural + deforestation)]

Table S5. Ratios between the different emission estimates of combustion-generated pollutants in the residential sector over Northern China between November and February. The inventories include Cheng et al. (Cheng et al., 2017) (Cheng), Multi-resolution Emission Inventory (MEIC) (Zhang et al., 2009), and Peking University inventory (PKU, the emission inventory adopted in this study (Shen et al., 2013)). Cheng et al. (Cheng et al., 2017) addressed the underestimation of fuel consumption in Northern China during the heating months and derived a correction factor for the residential sector emissions from the MEIC inventory (first row, Cheng/MEIC). This ratio is combined with the ratio between PKU and MEIC emissions (PKU/MEIC, next row) in order to estimate the correction factors for the PKU inventory (Cheng/PKU). Disregarding the very high ratio of the $PM_{2.5}$ emissions, the median correction factor for these pollutants is around 2.5. This value therefore multiplies the winter residential emissions in the regions shown in Figure S2 in the emission scenario E3. The MEIC dataset is available at http://www.meicmodel.org (last access: 16 March 2023).

	CO	NOx	PM_{10}	$PM_{2.5}$	OC	BC
Cheng/MEIC	2	1.40	3	14.7	1.59	2.7
PKU/MEIC	0.57	0.74	0.83	0.78	0.72	1.30
Cheng/PKU	3.51	1.89	3.37	18.85	2.21	2.07

Table S6: (a) Deposition velocities of anthracene ($v_{\text{dep,ANT}}$) and anthraquinone ($v_{\text{dep,AQ}}$) in unit of cm s^{-1} adopted in our model based on different land use types. (b) Allocation of the fraction of the three land surface categories, f_{veg} (covered by vegetation), f_{water} (by water) and f_{soil} (by bare soil) in each MOZART land use types.

(a)

Land use	Deposition velocity (cm/s)
**Anthracene ($v_{\text{dep,ANT}}$)**	
Land	0.24
Ocean	0.05
**Anthraquinone ($v_{\text{dep,AQ}}$)**	
1. Urban land	0.2
2. Agricultural land	0.3
3. Range land	0.15
4. Deciduous forest	0.3
5. Coniferous forest	0.3
6. Mixed forest including wetland	0.3
7. Water, both salt and fresh	0.16
8. Barren land, mostly desert	0.05
9. Non-forested wetland	0.1
10. Mixed agricultural and range land	0.3
11. Rocky open areas with low growing shrubs	0.05

(b)

MOZART land use types	f_{veg}	f_{water}	f_{soil}
1. Urban land	0.1	-	0.9
2. Agricultural land	1.0	-	-
3. Range land	0.7	-	0.3
4. Deciduous forest	1.0	-	-
5. Coniferous forest	1.0	-	-
6. Mixed forest including wetland	0.95	0.05	-
7. Water, both salt and fresh	-	1.0	-

8. Barren land, mostly desert	0.05	-	0.95
9. Non-forested wetland	0.1	0.9	-
10. Mixed agricultural and range land	0.9	-	0.1
11. Rocky open areas with low growing shrubs	0.1	-	0.9

Table S7. Summary of all simulation cases adopted in the present study. The assumptions are labelled as E1-3 for the emission scenarios, Y1-2 for the yield of anthraquinone formation from anthracene photo-oxidation and D1- 4 for the anthraquinone destruction rate.

Simulation	Description of case	Emission scenario	AQ production yield	AQ destruction time scale (overhead Sun)	With detailed PAH deposition
M1	High chemical production, low loss, highest seasonality in emissions, detailed deposition treatment	Enhanced seasonality + Enhanced winter emission (E3)	50% (Y1)	10 days (D1)	Yes
M2	High chemical production, highest loss, high seasonality in emissions, detailed deposition treatment	Enhanced seasonality relative to PKU-PAH inventory (E2)	50% (Y1)	20 minutes (D2)	Yes
M3	High chemical production, low loss, Reduced residential emissions from M2. high seasonality in emissions, detailed deposition treatment	Residential emissions of E2 reduced by 66%	50% (Y1)	10 days (D1)	Yes
S1	Low chemical production, high loss, low seasonality in emissions	Original PKU-PAH inventory (E1)	10% (Y2)	1 hour (D4)	No
S2	Low chemical production, high loss, high seasonality in emissions	Enhanced seasonality relative to PKU-PAH inventory (E2)	10% (Y2)	1 hour (D4)	No
S3	Low chemical production, very high loss, high seasonality in emissions	Enhanced seasonality relative to PKU-PAH inventory (E2)	10% (Y2)	40 minutes (D3)	No
S4	Low chemical production, low loss, low seasonality in emissions	Original PKU-PAH inventory (E1)	10% (Y2)	10 days (D1)	No
S5	Low chemical production, low loss, high seasonality in emissions	Enhanced seasonality relative to PKU-PAH inventory (E2)	10% (Y2)	10 days (D1)	No
S6	High chemical production, low loss, high seasonality in emissions	Enhanced seasonality relative to PKU-PAH inventory (E2)	50% (Y1)	10 days (D1)	No
S7	Low chemical production, very high loss, highest seasonality in emissions	Enhanced seasonality + Enhanced winter emission (E3)	10% (Y2)	40 minutes (D3)	No
S8	High chemical production, highest loss, highest seasonality in emissions	Enhanced seasonality + Enhanced winter emission (E3)	50% (Y1)	20 minutes (D2)	No

Table S8. Model performance against surface concentration measurements for the different simulation cases. Please refer to Table S7 for the simulation scenarios adopted for each simulation case and the following supplementary text for the evaluation methods.

Simulation	Description of case	Background		Non-background	
		NMB	*r*	*NMB*	*r*
M1	High chemical production, low loss, highest seasonality in emissions, detailed deposition treatment	**-0.07**	**0.69**	**-0.08**	**0.65**
M2	High chemical production, highest loss, high seasonality in emissions, detailed deposition treatment	-0.52	0.65	-0.54	0.59
S1	Low chemical production, high loss, low seasonality in emissions	-0.64	0.63	-0.63	0.49
S2	Low chemical production, high loss, high seasonality in emissions	-0.61	0.63	-0.62	0.56
S3	Low chemical production, very high loss, high seasonality in emissions	-0.58	0.64	-0.59	0.57
S4	Low chemical production, low loss, low seasonality in emissions	-0.43	0.66	-0.43	0.51
S5	Low chemical production, low loss, high seasonality in emissions	-0.42	0.67	-0.42	0.58
S6	High chemical production, low loss, high seasonality in emissions	-0.20	0.67	-0.20	0.57
S7	Low chemical production, very high loss, highest seasonality in emissions	-0.34	0.67	-0.37	0.62
S8	High chemical production, highest loss, highest seasonality in emissions	-0.27	0.66	-0.31	0.61

Table S9: Best-fit values of $K_{p,298}$ and Q_v from the 3 regression models in Figure S4 and the $K_{p,298}$ values from the Mackay and Octanol/air (Koa) models. N refers to the number of available measurements for the corresponding regression fit. The $K_{p,298}$ values from the latter two models are obtained from the EPI suite.

Regression/EPI Suite Model	$K_{p,298}$ ($m^3/\mu g$)	Q_v (kJ mol^{-1})	Norm of residues
fit obs (N=7)	0.0314	58.52	1.542
fit CAMS aerosols (N=27)	0.0148	43.61	3.642
fit CAMS PM10 (N=27)	0.0112	43.31	3.755
Mackay	0.000509	-	-
Octanol/air (Koa)	0.000627	-	-

Table S10: Best-fit values of $K_{p,298}$ and Q_v from the 3 regression models in Figure S7 and the $K_{p,298}$ values from the Mackay and Octanol/air (Koa) models. N refers to the number of available measurements for the corresponding regression fit. The $K_{p,298}$ values from the latter two models are obtained from the EPI suite. -

Regression/EPI Suite Model	$K_{p,298}$ ($m^3/\mu g$)	Q_v (kJ mol^{-1})	Norms of residues
fit obs (N=4)	9.04×10^{-5}	19.66	0.4104
fit CAMS aerosols (N=22)	5.24×10^{-4}	31.34	3.393
fit CAMS PM10 (N=22)	6.76×10^{-4}	30.8	3.32
Fit Simcik (N=40)	0.0014	28.51	3.393
Mackay	4.55×10^{-5}	-	-
Octanol/air (Koa)	8.71×10^{-6}	-	-

SI References

Alam, M. S., J. M. Delgado-Saborit, C. Stark & R. M. Harrison. 2013. Using atmospheric measurements of PAH and quinone compounds at roadside and urban background sites to assess sources and reactivity. *Atmospheric Environment* 77**:** 24-35.

Alam, M. S., J. M. Delgado-Saborit, C. Stark & R. Harrison. 2014. Investigating PAH relative reactivity using congener profiles, quinone measurements and back trajectories. *Atmos. Chem. Phys* 14**:** 2467-2477.

Alam, M. S., I. J. Keyte, J. Yin, C. Stark, A. M. Jones & R. M. Harrison. 2015. Diurnal variability of polycyclic aromatic compound (PAC) concentrations: relationship with meteorological conditions and inferred sources. *Atmospheric Environment* 122**:** 427-438.

Albinet, A., E. Leoz-Garziandia, H. Budzinski & E. Viilenave. 2006. Simultaneous analysis of oxygenated and nitrated polycyclic aromatic hydrocarbons on standard reference material 1649a (urban dust) and on natural ambient air samples by gas chromatography–mass spectrometry with negative ion chemical ionisation. *Journal of Chromatography A* 1121**:** 106-113.

Albinet, A., E. Leoz-Garziandia, H. Budzinski & E. Viilenave. 2007. Polycyclic aromatic hydrocarbons (PAHs), nitrated PAHs and oxygenated PAHs in ambient air of the Marseilles area (South of France): concentrations and sources. *Science of the Total Environment* 384**:** 280-292.

Albinet, A., E. Leoz-Garziandia, H. Budzinski, E. Villenave & J.-L. Jaffrezo. 2008. Nitrated and oxygenated derivatives of polycyclic aromatic hydrocarbons in the ambient air of two French alpine valleys: Part 1: Concentrations, sources and gas/particle partitioning. *Atmospheric Environment* 42**:** 43-54.

Atkinson, R., S. M. Aschmann, J. Arey, Z. Barbara & D. Schuetzle. 1989. Gas-phase atmospheric chemistry of 1-and 2-nitronaphthalene and 1, 4-naphthoquinone. *Atmospheric Environment (1967)* 23**:** 2679-2690.

Bardi, G., R. Gigli, L. Malaspina & V. Piacente. 1973. Vapor pressure and sublimation enthalpy of anthraquinone and of 1,5- and 1,8-dihydroxyanthraquinones. *Journal of Chemical & Engineering Data* 18**:** 126-130.

Bayona, J. M., M. Casellas, P. Fernandez, A. Solanas & J. Albaiges. 1994. Sources and seasonal variability of mutagenic agents in the Barcelona city aerosol. *Chemosphere* 29**:** 441-450.

Brasseur, G. P., D. A. Hauglustaine, S. Walters, P. J. Rasch, J. F. Müller, C. Granier & X. X. Tie. 1998. MOZART, a global chemical transport model for ozone and related chemical tracers: 1. Model description. *Journal of Geophysical Research: Atmospheres* 103**:** 28265-28289.

Castells, P., F. Santos & M. Galceran. 2003. Development of a sequential supercritical fluid extraction method for the analysis of nitrated and oxygenated derivatives of polycyclic aromatic hydrocarbons in urban aerosols. *Journal of Chromatography A* 1010**:** 141-151.

Cautreels, W., K. Van Cauwenberghe & L. Guzman. 1977. Comparison between the organic fraction of suspended matter at a background and an urban station. *Science of the Total Environment* 8**:** 79-88.

Cavanagh, J.-a. E. 2009. Ambient Concentrations of Polycyclic Aromatic Hydrocarbons (PAHs) in Christchurch, 2008-09. Environment Canterbury Regional Council.

Chang, K.-F., G.-C. Fang, C. Lu & H. Bai. 2003. imating PAH Dry Deposition by Measuring Gas and Particle Phase Concentrations in Ambient Air. *Aerosol and Air Quality Research* 3**:** 41-51.

Chen, W. & T. Zhu. 2014. Formation of nitroanthracene and anthraquinone from the heterogeneous reaction between NO2 and anthracene adsorbed on NaCl particles. *Environmental science & technology* 48**:** 8671-8678.

Cheng, M., G. Zhi, W. Tang, S. Liu, H. Dang, Z. Guo, J. Du, X. Du, et al. 2017. Air pollutant emission from the underestimated households' coal consumption source in China. *Science of the Total Environment* 580**:** 641-650.

Cho, A. K., E. Di Stefano, Y. You, C. E. Rodriguez, D. A. Schmitz, Y. Kumagai, A. H. Miguel, A. Eiguren-Fernandez, et al. 2004. Determination of four quinones in diesel exhaust particles, SRM 1649a, and atmospheric PM2. 5 special issue of aerosol science and technology on findings from the fine particulate matter supersites program. *Aerosol Science and Technology* 38**:** 68-81.

Chung, M. Y., R. A. Lazaro, D. Lim, J. Jackson, J. Lyon, D. Rendulic & A. S. Hasson. 2006. Aerosol-borne quinones and reactive oxygen species generation by particulate matter extracts. *Environmental science & technology* 40**:** 4880-4886.

Das, C. K. & N. S. Das. 1982. Oxidation of anthracene to anthraquinone in liquid-phase with an air/oxygen/nitric acid system. *Journal of Chemical Technology and Biotechnology* 32**:** 643-649.

Delgado-Saborit, J. M., M. S. Alam, K. J. G. Pollitt, C. Stark & R. M. Harrison. 2013. Analysis of atmospheric concentrations of quinones and polycyclic aromatic hydrocarbons in vapour and particulate phases. *Atmospheric Environment* 77**:** 974-982.

Delhomme, O., M. Millet & P. Herckes. 2008. Determination of oxygenated polycyclic aromatic hydrocarbons in atmospheric aerosol samples by liquid chromatography–tandem mass spectrometry. *Talanta* 74**:** 703-710.

Drotikova, T., A. M. Ali, A. K. Halse, H. C. Reinardy & R. Kallenborn. 2020. Polycyclic aromatic hydrocarbons (PAHs) and oxy-and nitro-PAHs in ambient air of the Arctic town Longyearbyen, Svalbard. *Atmospheric Chemistry and Physics* 20**:** 9997-10014.

Eiguren-Fernandez, A., A. H. Miguel, E. Di Stefano, D. A. Schmitz, A. K. Cho, S. Thurairatnam, E. L. Avol & J. R. Froines. 2008. Atmospheric distribution of gas-and particle-phase quinones in Southern California. *Aerosol Science and Technology* 42**:** 854-861.

Emmons, L. K., S. Walters, P. G. Hess, J.-F. Lamarque, G. G. Pfister, D. Fillmore, C. Granier, A. Guenther, et al. 2010. Description and evaluation of the Model for Ozone and Related chemical Tracers, version 4 (MOZART-4). *Geoscientific Model Development* 3**:** 43-67.

Finizio, A., D. Mackay, B. Terry & T. Harner. 1997. Octanol–Air Partition Coefficient as a Predictor of Partitioning of Semivolatile Organic Chemicals. *Atmospheric Environment* 31**:** 2289-2296.

Fraser, M. P., M. J. Kleeman, J. J. Schauer & G. R. Cass. 2000. Modeling the atmospheric concentrations of individual gas-phase and particle-phase organic compounds. *Environmental science & technology* 34**:** 1302-1312.

Giorgi, F. & W. L. Chameides. 1985. The rainout parameterization in a photochemical model. *Journal of Geophysical Research: Atmospheres* 90**:** 7872-7880.

Goulay, F., C. Rebrion-Rowe, J. L. L. Garrec, S. D. L. Picard, A. Canosa & B. R. Rowe. 2005. The reaction of anthracene with OH radicals: An experimental study of the kinetics between 58 and 470K. *The Journal of Chemical Physics* 122**:** 104308.

Guevara, M., O. Jorba, C. Tena, H. Denier Van Der Gon, J. Kuenen, N. Elguindi-Solmon, S. Darras, C. Granier, et al. 2020. CAMS-TEMPO: global and European emission temporal profile maps for atmospheric chemistry modelling. *Earth System Science Data Discussions***:** 1-60.

Harrison, R. M., M. S. Alam, J. Dang, I. Ismail, J. Basahi, M. A. Alghamdi, I. Hassan & M. Khoder. 2016. Relationship of polycyclic aromatic hydrocarbons with oxy (quinone) and nitro derivatives during air mass transport. *Science of the Total Environment* 572**:** 1175-1183.

Hodzic, A., S. Madronich, B. Aumont, J. Lee-Taylor, T. Karl, M. Camredon & C. Mouchel-Vallon. 2013. Limited influence of dry deposition of semivolatile organic vapors on secondary organic aerosol formation in the urban plume. *Geophysical Research Letters* 40**:** 3302-3307.

Huang, B., M. Liu, X. Bi, C. Chaemfa, Z. Ren, X. Wang, G. Sheng & J. Fu. 2014. Phase distribution, sources and risk assessment of PAHs, NPAHs and OPAHs in a rural site of Pearl River Delta region, China. *Atmospheric Pollution Research* 5**:** 210-218.

Inness, A., M. Ades, A. Agustí-Panareda, J. Barré, A. Benedictow, A.-M. Blechschmidt, J. J. Dominguez, R. Engelen, et al. 2019. The CAMS reanalysis of atmospheric composition. *Atmospheric Chemistry and Physics* 19**:** 3515-3556.

Janssens-Maenhout, G., M. Crippa, D. Guizzardi, M. Muntean, E. Schaaf, F. Dentener, P. Bergamaschi, V. Pagliari, et al. 2017. EDGAR v4.3.2 Global Atlas of the three major Greenhouse Gas Emissions for the period 1970-2012. *Earth Syst. Sci. Data Discuss.* 2017**:** 1-55.

Kallio, M., T. Hyötyläinen, M. Lehtonen, M. Jussila, K. Hartonen, M. Shimmo & M.-L. Riekkola. 2003. Comprehensive two-dimensional gas chromatography in the analysis of urban aerosols. *Journal of Chromatography A* 1019**:** 251-260.

Kelly, G. W., K. D. Bartle, A. A. Clifford & D. Scammells. 1993. Identification and Quantitation of Polycyclic Aromatic Compounds in Air Particulate and Diesel Exhaust Particulate Extracts by LC—GC. *Journal of chromatographic science* 31**:** 73-76.

Keyte, I. J., A. Albinet & R. M. Harrison. 2016. On-road traffic emissions of polycyclic aromatic hydrocarbons and their oxy-and nitro-derivative compounds measured in road tunnel environments. *Science of the Total Environment* 566**:** 1131-1142.

Kojima, Y., K. Inazu, Y. Hisamatsu, H. Okochi, T. Baba & T. Nagoya. 2010. Influence of secondary formation on atmospheric occurrences of oxygenated polycyclic aromatic hydrocarbons in airborne particles. *Atmospheric Environment* 44**:** 2873-2880.

Lee, Y. N. & X. Zhou. 1993. Method for the determination of some soluble atmospheric carbonyl compounds. *Environmental Science & Technology* 27**:** 749-756.

Li, W., C. Wang, H. Shen, S. Su, G. Shen, Y. Huang, Y. Zhang, Y. Chen, et al. 2015. Concentrations and origins of nitro-polycyclic aromatic hydrocarbons and oxy-polycyclic aromatic hydrocarbons in ambient air in urban and rural areas in northern China. *Environmental Pollution* 197**:** 156-164.

Lin, Y., Y. Ma, X. Qiu, R. Li, Y. Fang, J. Wang, Y. Zhu & D. Hu. 2015. Sources, transformation, and health implications of PAHs and their nitrated, hydroxylated, and oxygenated derivatives in PM2. 5 in Beijing. *Journal of Geophysical Research: Atmospheres* 120**:** 7219-7228.

Liu, Y., M. Sklorz, J. Schnelle-Kreis, J. Orasche, T. Ferge, A. Kettrup & R. Zimmermann. 2006. Oxidant denuder sampling for analysis of polycyclic aromatic hydrocarbons and their oxygenated derivates in ambient aerosol: Evaluation of sampling artefact. *Chemosphere* 62**:** 1889-1898.

Lyman, W. J., W. F. Reehl & D. H. Rosenblatt (eds.) 1990. *Handbook of chemical property estimation methods: Environmental behavior of organic compounds,* Washington, DC: American Chemical Society.

Lyu, R., Z. Shi, M. S. Alam, X. Wu, D. Liu, T. V. Vu, C. Stark, P. Fu, et al. 2019. Insight into the composition of organic compounds (≥ C 6) in PM 2.5 in wintertime in Beijing, China. *Atmospheric Chemistry and Physics* 19**:** 10865-10881.

Mackay, D., S. Paterson & W. H. Schroeder. 1986. Model describing the rates of transfer processes of organic chemicals between atmosphere and water. *Environmental Science & Technology* 20**:** 810-816.

Manchester-Neesvig, J. B., J. J. Schauer & G. R. Cass. 2003. The distribution of particle-phase organic compounds in the atmosphere and their use for source apportionment during the Southern California Children's Health Study. *Journal of the Air & Waste Management Association* 53**:** 1065-1079.

Manion, J. A., R. E. Huie, R. D. Levin, D. R. Burgess Jr, V. L. Orkin, W. Tsang, W. S. Mcgivern, J. W. Hudgens, et al. 2008. NIST chemical kinetics database. *NIST standard reference database.*

Maria Del Rosario Sienra, M. 2006. Oxygenated polycyclic aromatic hydrocarbons in urban air particulate matter. *Atmospheric Environment* 40**:** 2374-2384.

Mcgillen, M. R., W. P. L. Carter, A. Mellouki, J. J. Orlando, B. Picquet-Varrault & T. J. Wallington. 2020. Database for the kinetics of the gas-phase atmospheric reactions of organic compounds. *Earth Syst. Sci. Data* 12**:** 1203-1216.

Miet, K., A. Albinet, H. Budzinski & E. Villenave. 2014. Atmospheric reactions of 9, 10-anthraquinone. *Chemosphere* 107**:** 1-6.

Mirivel, G., V. Riffault & J.-C. Galloo. 2010. Simultaneous determination by ultra-performance liquid chromatography–atmospheric pressure chemical ionization time-of-flight mass spectrometry of nitrated and oxygenated PAHs found in air and soot particles. *Analytical and bioanalytical chemistry* 397**:** 243-256.

Müller, J.-F., T. Stavrakou, M. Bauwens, S. Compernolle & J. Peeters. 2018. Chemistry and deposition in the Model of Atmospheric composition at Global and Regional scales using Inversion Techniques for Trace gas Emissions (MAGRITTE v1.0). Part B. Dry deposition. *Geoscientific Model Development [preprint]*.

Nguyen, T. B., J. D. Crounse, A. P. Teng, J. M. S. Clair, F. Paulot, G. M. Wolfe & P. O. Wennberg. 2015. Rapid deposition of oxidized biogenic compounds to a temperate forest. *Proceedings of the National Academy of Sciences* 112**:** E392-E401.

Nicol, S., J. Dugay & M.-C. Hennion. 2001. Determination of oxygenated polycyclic aromatic compounds in airborne particulate organic matter using gas chromatography-tandem mass spectrometry. *Chromatographia* 53**:** S464-S469.

Niederer, M. 1998. Determination of polycyclic aromatic hydrocarbons and substitutes (nitro-, oxy-PAHs) in urban soil and airborne particulate by GC-MS and NCI-MS/MS. *Environmental Science and Pollution Research* 5**:** 209.

Offenberg, J., T. Kleindienst, M. Jaoui, M. Lewandowski & E. Edney. 2006. Thermal properties of secondary organic aerosols. *Geophysical Research Letters - GEOPHYS RES LETT* 330.

Pankow, J. F. 1994. An absorption model of the gas/aerosol partitioning involved in the formation of secondary organic aerosol. *Atmospheric Environment* 28**:** 189-193.

Park, S. S., M.-S. Bae, J. J. Schauer, Y. J. Kim, S. Y. Cho & S. J. Kim. 2006. Molecular composition of PM2. 5 organic aerosol measured at an urban site of Korea during the ACE-Asia campaign. *Atmospheric Environment* 40**:** 4182-4198.

Reddy, M. S., O. Boucher, N. Bellouin, M. Schulz, Y. Balkanski, J. L. Dufresne & M. Pham. 2005. Estimates of global multicomponent aerosol optical depth and direct radiative perturbation in the Laboratoire de Météorologie Dynamique general circulation model. *Journal of Geophysical Research: Atmospheres* 110.

Ringuet, J., E. Leoz-Garziandia, H. Budzinski, E. Villenave & A. Albinet. 2012. Particle size distribution of nitrated and oxygenated polycyclic aromatic hydrocarbons (NPAHs and OPAHs) on traffic and suburban sites of a European megacity: Paris (France). *Atmospheric Chemistry and Physics* 12**:** 8877–8887.

Rissanen, T., T. Hyötyläinen, M. Kallio, J. Kronholm, M. Kulmala & M.-L. Riekkola. 2006. Characterization of organic compounds in aerosol particles from a coniferous forest by GC–MS. *Chemosphere* 64**:** 1185-1195.

Schnelle-Kreis, J., I. Gebefügi, G. Welzl, T. Jaensch & A. Kettrup. 2001. Occurrence of particle-associated polycyclic aromatic compounds in ambient air of the city of Munich. *Atmospheric Environment* 35**:** S71-S81.

Schnelle-Kreis, J., M. Sklorz, A. Peters, J. Cyrys & R. Zimmermann. 2005. Analysis of particle-associated semi-volatile aromatic and aliphatic hydrocarbons in urban particulate matter on a daily basis. *Atmospheric Environment* 39**:** 7702-7714.

Schnelle-Kreis, J., M. Sklorz, J. Orasche, M. Stölzel, A. Peters & R. Zimmermann. 2007. Semi volatile organic compounds in ambient PM2. 5. Seasonal trends and daily resolved source contributions. *Environmental Science & Technology* 41**:** 3821-3828.

Shen, G. 2014. *Emission factors of carbonaceous particulate matter and polycyclic aromatic hydrocarbons from residential solid fuel combustions.* Springer-Verlag Berlin Heidelberg.

Shen, H., Y. Huang, R. Wang, D. Zhu, W. Li, G. Shen, B. Wang, Y. Zhang, et al. 2013. Global atmospheric emissions of polycyclic aromatic hydrocarbons from 1960 to 2008 and future predictions. *Environmental science & technology* 47**:** 6415-6424.

Shimizu, T., S. Ohkubo, M. Kimura, I. Tabata & T. Hori. 1987. The vapour pressures and heats of sublimation of model disperse dyes. *Journal of the Society of Dyers and Colourists* 103**:** 132-137.

Shimmo, M., P. Anttila, K. Hartonen, T. Hyötyläinen, J. Paatero, M. Kulmala & M.-L. Riekkola. 2004. Identification of organic compounds in atmospheric aerosol particles by on-line supercritical fluid extraction–liquid chromatography–gas chromatography–mass spectrometry. *Journal of Chromatography A* 1022**:** 151-159.

Simcik, M. F., T. P. Franz, H. Zhang & S. J. Eisenreich. 1998. Gas-Particle Partitioning of PCBs and PAHs in the Chicago Urban and Adjacent Coastal Atmosphere: States of Equilibrium. *Environmental Science & Technology* 32**:** 251-257.

Sklorz, M., J.-J. Briedé, J. Schnelle-Kreis, Y. Liu, J. Cyrys, T. M. De Kok & R. Zimmermann. 2007. Concentration of oxygenated polycyclic aromatic hydrocarbons and oxygen free radical formation from urban particulate matter. *Journal of Toxicology and Environmental Health, Part A* 70**:** 1866-1869.

Smit, A., F. Van Den Berg & M. Leistra 1997. Estimation method for the volatilization of pesticides from fallow soil. DLO Winand Staring Centre.

Smit, A., M. Leistra & F. Van Den Berg 1998. Estimation method for the volatilization of pesticides from plants. DLO Winand Staring Centre.

Souza, K. F., L. R. Carvalho, A. G. Allen & A. A. Cardoso. 2014. Diurnal and nocturnal measurements of PAH, nitro-PAH, and oxy-PAH compounds in atmospheric particulate matter of a sugar cane burning region. *Atmospheric environment* 83**:** 193-201.

Takekawa, H., H. Minoura & S. Yamazaki. 2003. Temperature dependence of secondary organic aerosol formation by photo-oxidation of hydrocarbons. *Atmospheric Environment* 37**:** 3413-3424.

Tasdemir, Y. & F. Esen. 2007. Dry deposition fluxes and deposition velocities of PAHs at an urban site in Turkey. *Atmospheric Environment* 41**:** 1288-1301.

Tilmes, S. 2016. GEOS5 Global Atmosphere Forcing Data. Research Data Archive at the National Center for Atmospheric Research, Computational and Information Systems Laboratory.

Tomaz, S., J.-L. Jaffrezo, O. Favez, E. Perraudin, E. Villenave & A. Albinet. 2017. Sources and atmospheric chemistry of oxy-and nitro-PAHs in the ambient air of Grenoble (France). *Atmospheric Environment* 161**:** 144-154.

Tsapakis, M., E. Lagoudaki, E. G. Stephanou, I. G. Kavouras, P. Koutrakis, P. Oyola & D. Von Baer. 2002. The composition and sources of PM2. 5 organic aerosol in two urban areas of Chile. *Atmospheric Environment* 36**:** 3851-3863.

U.S. National Library of Medicine 2006. Hazardous Substances Data Bank. Anthraquinone (CAS No. 84-65-1).

United States Environmental Protection Agency 2011. Estimation Program Interface (EPI) Suite. US EPA Washington, DC.

Valavanidis, A., K. Fiotakis, T. Vlahogianni, E. B. Bakeas, S. Triantafillaki, V. Paraskevopoulou & M. Dassenakis. 2006. Characterization of atmospheric particulates, particle-bound transition metals and polycyclic aromatic hydrocarbons of urban air in the centre of Athens (Greece). *Chemosphere* 65**:** 760-768.

Wang, J., S. S. H. Ho, R. Huang, M. Gao, S. Liu, S. Zhao, J. Cao, G. Wang, et al. 2016. Characterization of parent and oxygenated-polycyclic aromatic hydrocarbons (PAHs) in Xi'an, China during heating period: An investigation of spatial distribution and transformation. *Chemosphere* 159**:** 367-377.

Wang, L., R. Atkinson & J. Arey. 2007. Formation of 9, 10-phenanthrenequinone by atmospheric gas-phase reactions of phenanthrene. *Atmospheric Environment* 41**:** 2025-2035.

Wang, R., S. Tao, P. Ciais, H. Shen, Y. Huang, H. Chen, G. Shen, B. Wang, et al. 2013. High-resolution mapping of combustion processes and implications for CO2 emissions. *Atmos. Chem. Phys* 13**:** 5189-5203.

Wang, W., N. Jariyasopit, J. Schrlau, Y. Jia, S. Tao, T.-W. Yu, R. H. Dashwood, W. Zhang, et al. 2011. Concentration and photochemistry of PAHs, NPAHs, and OPAHs and toxicity of PM2. 5 during the Beijing Olympic Games. *Environmental science & technology* 45**:** 6887-6895.

Wei, C., Y. Han, B. a. M. Bandowe, J. Cao, R.-J. Huang, H. Ni, J. Tian & W. Wilcke. 2015. Occurrence, gas/particle partitioning and carcinogenic risk of polycyclic aromatic hydrocarbons and their oxygen and nitrogen containing derivatives in Xi'an, central China. *Science of the total environment* 505**:** 814-822.

Wei, S., B. Huang, M. Liu, X. Bi, Z. Ren, G. Sheng & J. Fu. 2012. Characterization of PM2. 5-bound nitrated and oxygenated PAHs in two industrial sites of South China. *Atmospheric Research* 109**:** 76-83.

Wesely, M. L. 2007. Parameterization of surface resistances to gaseous dry deposition in regional-scale numerical models. *Atmospheric Environment* 41**:** 52-63.

Wingfors, H., L. Hägglund & R. Magnusson. 2011. Characterization of the size-distribution of aerosols and particle-bound content of oxygenated PAHs, PAHs, and n-alkanes in urban environments in Afghanistan. *Atmospheric Environment* 45**:** 4360-4369.

Yalkowsky, S. H., Y. He & P. Jain. 2016. *Handbook of aqueous solubility data.* CRC press.

Yassaa, N., B. Y. Meklati, A. Cecinato & F. Marino. 2001. Organic aerosols in urban and waste landfill of Algiers metropolitan area: occurrence and sources. *Environmental science & technology* 35**:** 306-311.

Zeng, M., Z. Liao & L. Wang. 2020. Atmospheric oxidation of gaseous anthracene and phenanthrene initiated by OH radicals. *Atmospheric Environment***:** 117587.

Zhang, Q., D. G. Streets, G. R. Carmichael, K. B. He, H. Huo, A. Kannari, Z. Klimont, I. S. Park, et al. 2009. Asian emissions in 2006 for the NASA INTEX-B mission. *Atmos. Chem. Phys.* 9**:** 5131-5153.

Zhang, Y. & S. Tao. 2008. Seasonal variation of polycyclic aromatic hydrocarbons (PAHs) emissions in China. *Environmental pollution* 156**:** 657-663.

Zhong, Q., Y. Huang, H. Shen, Y. Chen, H. Chen, T. Huang, E. Y. Zeng & S. Tao. 2017. Global estimates of carbon monoxide emissions from 1960 to 2013. *Environmental Science and Pollution Research* 24**:** 864-873.

Zielinska, B., J. Sagebiel, J. D. Mcdonald, K. Whitney & D. R. Lawson. 2004. Emission rates and comparative chemical composition from selected in-use diesel and gasoline-fueled vehicles. *Journal of the Air & Waste Management Association* 54**:** 1138-1150.
